# Optical Selection of Rotational and Parity-Resolved States for Rotationally Inelastic Scattering: NO(X^2^Π_1/2_, *v* = 1, *j* = 1.5*e*) with Ar and CH_4_

**DOI:** 10.1021/acs.jpca.5c08389

**Published:** 2026-03-09

**Authors:** Martin Fournier, Rebecca G. Cameron, Kenneth G. McKendrick, Matthew L. Costen

**Affiliations:** Institute of Chemical Sciences, School of Engineering and Physical Sciences, 3120 Heriot-Watt University, Edinburgh EH14 4AS, United Kingdom

## Abstract

We present new experimental measurements of rotationally inelastic scattering of vibrationally excited NO­(X^2^Π) with Ar and CH_4_. A molecular beam of NO was prepared in a single rotational and parity-resolved state, *j* = 1.5 *F*_1_ *e*, in the *v* = 1 vibrational level using mid-infrared radiation from a distributed feedback quantum cascade laser. Following collision with a crossed molecular beam of Ar or CH_4_, rotationally excited NO­(X, *v* = 1) in the isolated final rotational states *j′* = 4.5 *F*_1_ *f* and *j′* = 10.5 *F*_1_ *f* was detected by 1 + 1′ resonance-enhanced multiphoton ionization coupled with velocity-map imaging. Differential cross sections and rotational angular momentum polarization moments for inelastic scattering with Ar are in excellent, near-quantitative agreement with quantum scattering predictions on a literature potential energy surface. Images for scattering from CH_4_ for both final states show clear evidence of significant rotational excitation in the CH_4_. Overall, a negative correlation is observed in the NO–CH_4_ rotational excitation, with higher average CH_4_ rotational energy for final NO *j*′ = 4.5 than for *j′* = 10.5. For NO *j′* = 10.5, higher rotational energies of CH_4_ are surprisingly correlated with forward hemisphere scattering, while lower CH_4_ rotation is correlated with backward hemisphere scattering. These measurements demonstrate the importance of the preparation of an initial rotational and parity-selected state, and the varied and surprising dynamics that remain underexplored in molecule–molecule inelastic scattering.

## Graphical abstract

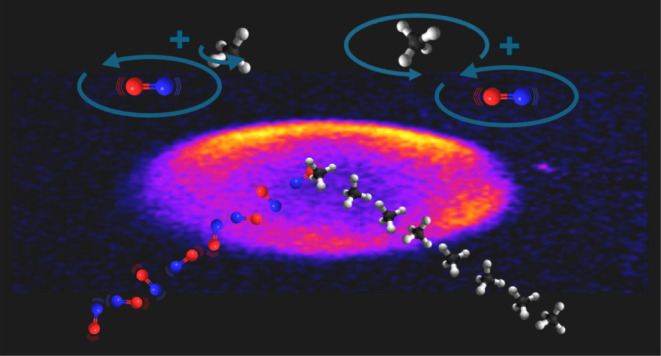

## Introduction

Rotational energy transfer (RET) is ubiquitous in the gas phase and important in the chemical evolution of widely varied environments including combustion, planetary atmospheres, and astrophysical molecular clouds. As well as this practical importance, the relative simplicity of energy transfer in comparison to reaction has resulted in its use as a test of theory, both in the electronic structure calculations that underpin potential energy surfaces (PESs) and in the scattering theory methods that use PESs.

For diatom–atom scattering, a detailed understanding of the dynamics has been established, including a range of interference and resonance effects, and near-quantitative agreement between experiment and theory is achievable. The NO­(X^2^Π) + rare gas systems have been the central piece of this effort, with crossed molecular beams combined with velocity-map ion imaging (CMB-VMI) as the most important experimental methodology.^1–10^

More recently, attention has turned to molecule–molecule collisions, where the additional degrees of freedom introduce new complexity in experiment and theory, and most importantly in the observed dynamics. A particular focus to date has been the ability of CMB-VMI experiments to measure rotational-state-correlated differential cross sections (DCSs), i.e., the scattering angle distribution for a product rotational state for one molecule (e.g., NO) in coincidence with a particular rotational state of the other molecule (e.g., O_2_). Focusing just on NO, measurements and scattering calculations of these correlated DCSs have been made for the diatom–diatom systems NO­(X) + O_2_, CO, and D_2_,^11–15^ and have more recently been extended to NO­(X) + ND_3_.^16^

Selection of an isolated initial rotational state has been central to the success of both the NO­(X) + rare gas and NO­(X) + molecule studies. This is particularly complicated in NO­(X), as the ^2^Π electronic character results not only in spin–orbit coupling to yield two spin–orbit manifolds, ^2^Π_1/2_ (*F*_1_) and ^2^Π_3/2_ (*F*_2_), but also in each rotational level, *j*, being split into two states with opposite rotational parities. These are the Λ-doublets, conventionally labeled *e* and *f*, which are very closely spaced energetically; for *j* = 0.5 in the lower spin–orbit manifold, *F*_1_, the splitting is 0.0119 cm^–1^.^17^ In a molecular beam expansion, these two parity states will be equally populated, and additional experimental measures are required to isolate a single state. One approach that has been applied by multiple groups is state selection using the Stark effect.^18^ The most straightforward implementation is the use of static electric fields in a hexapole, which acts as a filter that selects “low-field seeking” states. For NO­(X) *j* = 0.5 *F*_1_, this is the *f*-lambda doublet. CMB-VMI studies of inelastic collisions of NO­(X) with rare gases using hexapole state selection were pioneered by Stolte and coworkers and have subsequently been systematically studied by Brouard and coworkers.^1–4,19–28^ Time-varying electric fields can provide much greater initial state control, with Stark deceleration adding precise velocity selection, albeit at the cost of much greater experimental complexity. Van de Meerakker and coworkers have demonstrated how the very high resolution of both collision energy and scattering angle it provides can be used to measure diffractive oscillations, low-energy scattering resonances, and correlated rotational-state distributions.^7–13,15,16,29^

Optical excitation presents an alternative approach to the selection of a single rotational state. We have previously demonstrated this approach in CMB-VMI studies of rotationally inelastic collisions of electronically excited NO­(A^2^Σ^+^) with a range of rare gas and molecular collision partners.^30–36^ These experiments used absorption of a single ultraviolet-wavelength photon to prepare NO­(A, *v* = 0) in the single isolated *j* = 0.5 *f*_1_ spin-rotation resolved state, facilitating both high-resolution scattering measurements of DCSs and product rotational angular momentum polarization, and their interpretation through time-independent quantum scattering (QS) calculations. Optical state selection can also provide a degree of flexibility in the prepared state, albeit constrained by the initial molecular-beam rotational distribution and spectroscopic selection rules. In the NO­(A) experiments, we were also able to use the ^S^R_21_(0.5) transition to prepare the *j* = 1.5*f*_2_ spin-rotation resolved state with a well-defined angular momentum orientation, and hence study a 4-vector correlation between initial and final rotational angular momentum and direction.^37^ Further control over state preparation is possible with more complicated optical schemes. Stimulated-emission pumping (SEP) has been used by Suits and coworkers to prepare NO­(X) for rotationally inelastic scattering in a range of isolated ro-vibrational states.^38–44^ The additional spectroscopic flexibility provided by the pump and dump steps in SEP enables the production not only of highly vibrationally excited NO­(X), e.g., *v* = 10, but also rotationally or spin–orbit excited NO­(X). This enables tests of theory in regimes beyond those generally available in conventional CMB experiments.

An experimentally simpler approach to vibrational excitation and state selection is the use of single-photon infrared (IR) excitation. Pulsed tunable infrared lasers have a long history of use in energy transfer experiments, although they have not been applied in CMB-VMI experiments to date. However, the commercial availability of continuous-wave mid-IR quantum cascade lasers (QCLs) with narrow line widths (<5 MHz) and high powers (e.g., 100 mW) has recently provided an alternative, relatively inexpensive method for state preparation. Van de Meerakker and coworkers demonstrated the power of this approach in low-collision-energy scattering of NO­(X, *v* = 1, *j* = 1.5 *F*_1_ *f*) by He.^8^ The mid-IR radiation crossed the NO molecular beam at the exit of a Stark decelerator, pumping NO from the Stark-controlled and selected *v* = 0, *j* = 0.5 *F*_1_ *f* state to *v* = 1, *j* = 1.5 *F*_1_ *f*. The prepared NO subsequently crossed a beam of He at a 5° angle. The rotational energy released in de-excitation collisions to the final state *v* = 1, *j* = 0.5 *F*_1_ *e* or *f* enabled the accurate measurement of DCSs at precise collision energies in the 0.4 to 6 cm^–1^ range that corresponded to scattering resonances.

However, a Stark decelerator is not a requirement for this QCL-based mid-IR approach to state preparation. In this paper, we present a demonstration of the measurement of rotationally inelastic scattering dynamics of NO­(X) with both Ar and CH_4_, with the NO prepared in the *v* = 1, *j* = 1.5 *F*_1_ *e* initial level using mid-IR excitation by a QCL.

The NO­(X, *v* = 0) + Ar system has been very extensively studied by both experiment and theory, as described above, and is used here as a known test case to demonstrate our control and understanding of state preparation and product-state detection. Our product-state detection is sensitive to the polarization of the rotational angular momentum, defined with respect to the scattering plane formed by initial and final relative velocity, the ***k***-***k′*** plane. This is a well-understood property of rotationally inelastic scattering, for which the NO­(X) + rare gas systems have again stood as a test case.^3–5,22,45,46^ We describe the alignment of the product angular momentum in terms of three alignment moments, each of which is a function of scattering angle, $A_{0}^{\left(2\right)}\left(\theta \right)$ , $A_{1+}^{\left(2\right)}\left(\theta \right)$ and $A_{2+}^{\left(2\right)}\left(\theta \right)$ .^30,31,34^ These alignment moments, together with the DCS, may be determined from analysis of pairs of scattering images recorded using probe light that is linearly polarized either in the image plane or perpendicular to the image plane. The alignment moments may also be directly calculated from theory. The simplest model that predicts product angular momentum polarization is the kinematic apse (KA) conservation model, which has proved very successful at predicting product polarization in NO­(X) + Rg systems.^1,3,45^ KA conservation arises in classical impulsive collisions involving hard-shell PESs, where the angular momentum transferred to the diatomic molecule must lie perpendicular to the axis of linear-momentum transfer, defined as the kinematic apse ***a***_*k*_ = (***k*** ′ – ***k***)/(| ***k*** ′ – ***k*** |). The alignment moments that are predicted have no dependence on the PES and instead depend purely on the kinematics of the collision and the scattering angle. The alignment moments may also be calculated using more sophisticated classical methods, e.g., quasi-classical trajectories on accurate PESs, and by quantum scattering methodologies. We present experimental DCSs and product rotational angular momentum polarization moments for NO­(X, *v* = 1) scattering with Ar and compare them to the results of QS calculations on a literature *ab initio* PES.

The NO­(X) + CH_4_ system is much less well understood but is conceptually interesting as a prototypical diatom + spherical rotor collision system. There is a single previous CMB-VMI measurement of rotationally inelastic scattering in this system and no applicable scattering theory.^47^ That study did not apply any additional state selection beyond the initial expansion cooling provided by the molecular beam. The initial state(s) were hence a distribution of low-*j* in *v* = 0, *F*_1_ with equal populations of the *e* and *f* parities. However, despite these limitations, some surprising scattering dynamics were reported, specifically an anticorrelation in NO and CH_4_ rotational excitation, i.e., low-*j* NO correlated with high-*j* CH_4_ and *vice versa*. This is the opposite of the rotation–rotation correlations observed in NO + linear molecule collisions.^11–15,29,33,35,36^ We have therefore undertaken measurements on this system, with the addition of complete initial NO state selection, polarization-sensitive and near-ionization-threshold probing, and with lower background and higher signal-to-noise. We present DCSs for different product NO­(*v* = 1, *j′*) states as a function of the internal energy transferred to CH_4_ and discuss them in the context of the previous measurements and available theory.

## Experimental Methods

The experiments were performed in a CMB-VMI apparatus, adapted from one used to study rotationally inelastic scattering of NO­(A^2^Σ^+^) and previously described in detail.^30–37^ The molecular beam sources and central scattering chamber were unchanged, but the ion optics, time-of-flight tube, and detector were replaced. The new ion optics design was based on one introduced by Tkac et al.,^48^ itself based on the design proposed by Lin et al., capable of DC slice imaging if desired.^49,50^ The ion optics mechanical design and the associated electric field simulations (SIMION) are shown in the Supporting Information (SI) Section S.1. The 21 electrodes were 80 mm in outside diameter, with 5 of the electrodes independently biased by a precision HV power supply (ISEG GmbH, EHS-80–60p), and the remainder connected by in-vacuum resistors. The ion optics were calibrated by multiphoton dissociation and ionization of O_2_ at 224.999 nm,^51,52^ with additional measurements of NO­(X^2^Π, *v* = 1, *j*′) products from 325 nm photodissociation of NO_2_ performed to accurately determine the speed-pixel scaling ratio at low fragment kinetic energies. Images from these calibration experiments at a range of repeller voltages for both “crush” and dc-slicing, and the resulting speed-pixel calibrations are presented in SI Section S.2. For the experiments presented here, +600 V was applied to the repeller electrode of the ion optics, resulting in a speed-pixel ratio of 2.89 ± 0.02 ms^–1^ pixel^–1^. Ions were accelerated up a 1 m flight path to a 75 mm diameter detector consisting of a pair of microchannel plates and a phosphor screen connected to a fiber-bundle vacuum feedthrough (Photonis, APD 2PS 75/12/10/8). The output surface of the fiber bundle was imaged using a CMOS camera (Basler, a2A1920–160umBAS). The detector gain was gated by raising the voltage applied to the rear MCP by 500 V (Photek, GM-MCP-2). A minimum gate width of 20 ns is achievable, sufficient to implement dc-slicing;^50^ however, for the experiments presented here, a wider gate of 1 μs was used to image the entire NO Newton sphere. This “crush” imaging is necessary here to allow the determination of the product rotational angular momentum alignment moments, two of which, $A_{1+}^{\left(2\right)}\left(\theta \right)$ and $A_{2+}^{\left(2\right)}\left(\theta \right)$ , depend on the azimuthal scattering angle and hence the out-of-plane scattering that would not be detected in dc-slicing.^3^

In scattering experiments, the molecular beams crossed at right angles in the center of the velocity-mapping region of the ion optics. One beam contained NO (BOC, 99.998%) seeded (10%) in Ar (BOC, 99.998%), produced from a 3 bar backing pressure. The other beam was of either pure Ar or pure CH_4_ (BOC, 99.995%), both from 5 bar backing pressure. The resulting NO molecular beam had a Gaussian speed distribution with a mean of 615 ms^–1^ and full width at half-maximum (fwhm) of 43 ms^–1^, as determined by direct imaging of the mid-IR prepared NO­(*v* = 1, *j* = 1.5 *F*_1_ *e*). The Ar and CH_4_ beams had mean speeds of 601 ms^–1^ and 1110 ms^–1^, and fwhm of 43 ms^–1^ and 166 ms^–1^, respectively, measured by imaging a trace concentration (<0.05%) of NO­(X, *v* = 0) entrained in these gases. This resulted in Gaussian distributions for the center-of-mass collision energies with a mean 530 cm^–1^ and fwhm of 53 cm^–1^ for Ar, and mean 704 cm^–1^ with fwhm of 162 cm^–1^ for CH_4_. The CH_4_ rotational distribution was not determined, but literature measurements of pure-CH_4_ expansions have demonstrated good rotational cooling, with only significant population in the *j* = 0, 1, and 2 levels, all of which must be populated assuming spin symmetry conservation in the expansion.^53^ We have therefore assumed that only the *j* = 0 (meta), *j* = 1 (ortho), and *j* = 2 (para) levels have significant population.

NO­(X^2^Π) in the molecular beam was excited from the ground (*v* = 0, *F*_1_, *j* = 0.5*e*) state to the (*v* = 1, *F*_1_, *j* = 1.5*e*) state using a continuous-wave mid-infrared distributed-feedback quantum-cascade laser (DFB-QCL, Thorlabs, QD5250C2). The DFB-QCL was mounted in a thermoelectrically cooled temperature-stabilized mount (Thorlabs, LDMC20) connected to a temperature and current controller (Thorlabs, ITC4002QCL). The DFB-QCL produced single-frequency (see Results section for measurement of the effective bandwidth over typical experimental time scales) light in the frequency range 1879–1883 cm^–1^, with a maximum optical power of 110 mW at 1881 cm^–1^ in a 3 mm diameter beam. The mid-infrared beam crossed the NO/Ar molecular beam at right angles ≈5 mm upstream of the collision region.

After collision, NO­(X^2^Π, *v* = 1) was detected via 1 + 1′ REMPI using the NO­(A^2^Σ^+^-X^2^Π) transition on the (0,1) band around 235 nm for the resonant step, and ionization of the resulting A-state with 325 nm light. This introduces a photoionization recoil to the NO^+^ cation of 2.0 ms^–1^. The probe and ionization laser beams crossed at right angles in the center of the molecular beam crossing region. The probe laser beam was 2 mm in diameter, with a typical pulse energy of 100 μJ, and the ionization laser beam was 3 mm in diameter with a typical pulse energy of 3 mJ. The probe beam passed through a photoelastic modulator (Hinds Instruments, PEM-90), which allowed the switching of the probe polarization between two experimental geometries, horizontal (H), in which the electric vector of the probe light lies in the plane containing the two molecular beams, which is also the image plane, and vertical (V) in which the electric vector is perpendicular to the image plane.

Scattering images were recorded for two different final states, *j′* = 4.5 *F*_1_ *f* and *j′* = 10.5 *F*_1_ *f*, using the relevant ^S^R_21_ branch transitions, with each of the two collision partners. These two product states involve the transfer of 35 cm^–1^ and 194 cm^–1^ of the collision energy into rotation of NO, respectively. In both cases, multiple independent experiments were performed, with Ar and CH_4_ measurements interleaved on the same day. In each measurement, images were recorded in a repeating, alternating sequence of H and V, foreground and background. Both molecular beams were present for the foreground images of the scattering, while for the background images, the Ar or CH_4_ molecular beam was delayed by 1 ms (and hence was effectively absent for the purposes of scattering). The probe laser wavelength was scanned across the complete Doppler width of the probe transition 3 times during each measurement.

## Theoretical Methods

Close-coupled quantum scattering (QS) calculations for the NO­(X)+Ar system were performed using Hibridon 5.1, using the CCSD­(T) potential energy surface calculated by Alexander.^54,55^ Note that this surface was calculated for a fixed NO bond length, *r* = 1.15077 Å, corresponding to the equilibrium length for *v* = 0. The average bond length for *v* = 1 is only ≈0.5% larger than for *v* = 0, reflecting the strong bonding in NO.^56^ Calculations were performed at 22 total energies from 430 to 640 cm^–1^, in 10 cm^–1^ increments. NO­(X) was treated as a rigid rotor with the *v* = 1 rotational constants: *B* = 1.67853 cm^–1^; *A* = 122.9123 cm^–1^; *p* = 1.205 × 10^–2^ cm^–1^; and *q* = 1.10 × 10^–4^ cm^–1^.^56^ Scattering calculations were performed for total angular momentum, *J*_tot_, up to 200.5, with a rotational basis of states up to *j* = 20.5, and propagation from 3.5 to 40 bohr. Differential cross sections and the scattering angle-dependent rotational angular momentum polarization moments $A_{0}^{\left(2\right)}\left(\theta \right)$ , $A_{1+}^{\left(2\right)}\left(\theta \right)$ , and $A_{2+}^{\left(2\right)}\left(\theta \right)$ were determined from the resulting scattering matrices for each total energy and were then averaged over the experimental collision energy distribution.

## Results

Figure 1 shows the results of two different spectroscopic measurements that characterize the initial state preparation. These measurements were made with a single molecular beam containing 0.1% NO in Ar, and with greatly reduced 1 + 1′ REMPI laser fluences, to avoid saturation of the MCP detector. In the upper panel, we show mid-IR action spectra, recorded by fixing the UV probe laser either on the A-X­(0,1) R_11_(1.5) transition that probes purely *j* = 1.5 *F*_1_ *e*, or on the ^S^R_21_(1.5) transition that probes purely *j* = 1.5 *F*_1_ *f*, and then scanning the QCL frequency by varying the QCL temperature in steps of 1 mK at a fixed current of 380 mA, with the mid-IR power attenuated to 35 mW using a combination of a waveplate and a MgF_2_ Rochon polarizer (Thorlabs WPLH05M-5300 and RPM10). Each step took 30 s to acquire, averaging 300 probe laser pulses. When probing the *f* Λ-doublet, NO^+^ ions are only detected in the mid-IR frequency range shown by the red line. Similarly, when probing the *e* Λ-doublet, NO^+^ ions are only detected in the range shown by the blue line. The spectra are generally in good agreement with the hyperfine-resolved assignments in the HITRAN database, shown at the top of the panel, where the two scales have been aligned to achieve agreement between the *F″* = 0.5 - *F′* = 1.5 and *F″* = 1.5 - *F′* = 0.5 transitions for the *f* Λ-doublet, where *F* is the total angular momentum resulting from coupling of the ^14^N nuclear spin *I* = 1 with rotational angular momentum *j*.^17^ There are disagreements in the positions of individual transitions of up to 10 MHz, and repeated wavelength scans acquired over several hours produced similar spectra that resolved the hyperfine levels in the *f* Λ-doublet with variations in the apparent line positions up to 20 MHz. We can use the action spectra to estimate the effective frequency spread of the QCL over the time taken to probe a single hyperfine transition, which was approximately 5 min. The isolated transitions of the *f* Λ-doublet have a near-Gaussian line shape with a fwhm of ≈20 MHz. Homogeneous broadening of the line will be dominated by the ca. 5 μs transit time of NO through the laser beam, resulting in a line width of <1 MHz. Inhomogeneous Doppler broadening can be estimated from the transverse width of the NO beamspot, consistent with a fwhm of ≈4 MHz. We therefore believe that the majority of the effective short-term bandwidth arises from frequency instability in the QCL output, with contributions from both the current and temperature control. Slower drifts in either current or temperature control then result in the variations in measured line position within and between scans.

**Figure 1:**
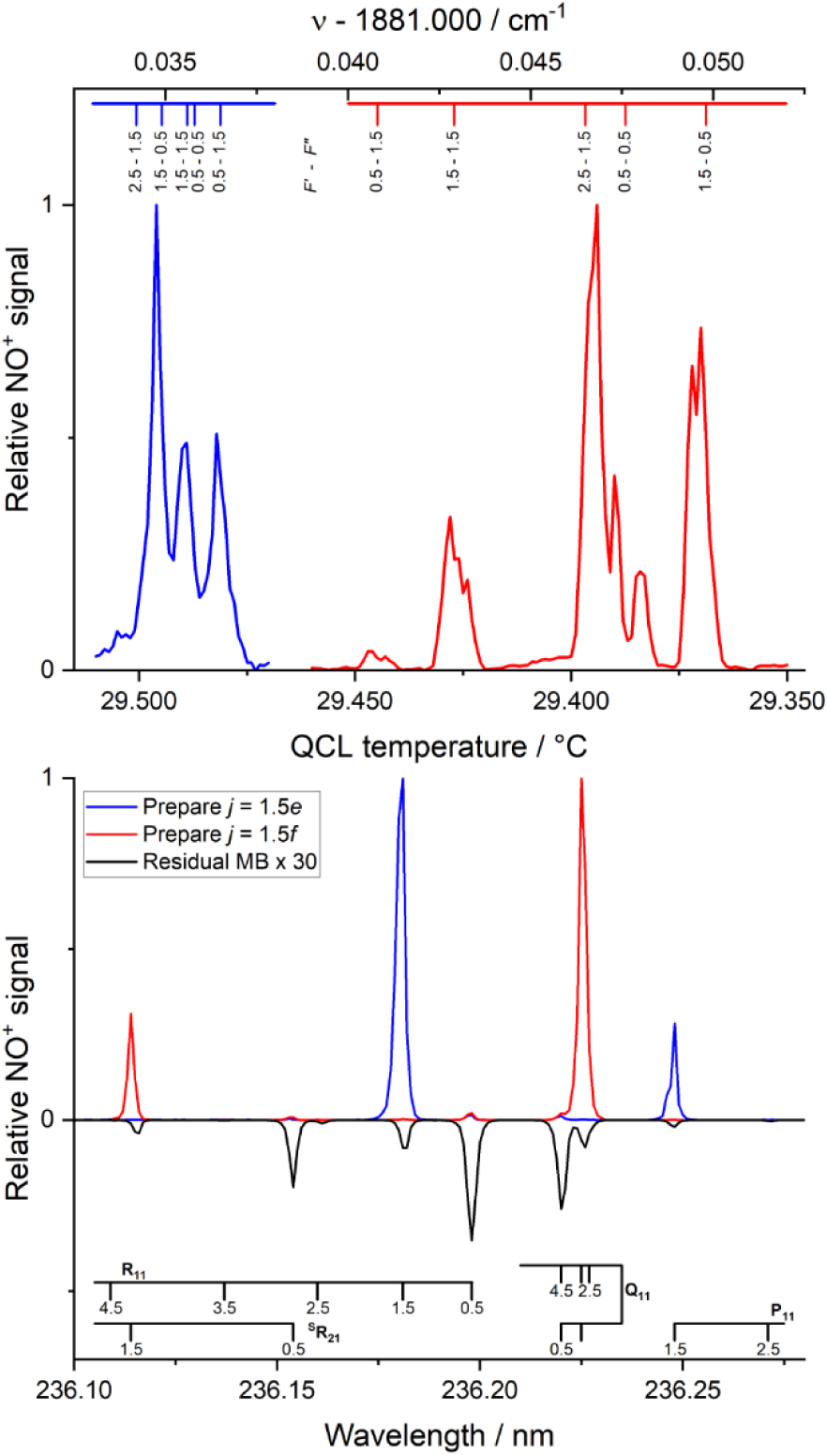
Upper panel: Hyperfine-resolved mid-IR action spectrum of the NO *v* = 0 → 1, *j* = 0.5 *F*_1_ *e*/*f* → *j* = 1.5 *F*_1_ *e*/*f* transitions. Blue line, detecting NO *v* = 1, *j* = 1.5 *F*_1_ *e.* Red line, detecting NO *v* = 1, *j* = 1.5 *F*_1_ *f*. Lower panel: (1 + 1′) REMPI spectra of NO taken on the A^2^Σ^+^-X^2^Π (0,1) band, with rotational assignments. Red line, with mid-IR preparation of *v* = 1, *j* = 1.5 *F*_1_ *f*. Blue line, with mid-IR preparation of *v* = 1, *j* = 1.5 *F*_1_ *e*. Black inverted line, residual *v* = 1 NO present in the molecular beam in the absence of mid-IR preparation, multiplied by a factor of 30.

In the lower panel of Figure 1, the QCL has been used to excite either the *e* Λ-doublet, nominally via the *j* = 0.5*e*, *F* = 1.5 → *j* = 1.5*e*, *F* = 2.5 transition at 1881.0342 cm^–1^ (blue line), or the *f* Λ-doublet nominally via the *j* = 0.5*f*, *F* = 1.5 → *j* = 1.5*f*, *F* = 2.5 transition at 1881.0465 cm^–1^ (red line), and the probe laser has been scanned to record a 1 + 1′ REMPI spectrum of the NO A-X­(0,1) bandhead region. Large signals are observed when resonant with the prepared *j* = 1.5 *e* or *f* level in each case. Very small residual signals from either *j* = 1.5 *e* or *f* are observed when preparing the other lambda-doublet, with a signal enhancement through the mid-IR excitation of ≈500. Additional small signals are observed, which primarily arise from *j* = 0.5 *e* and *f*. Without mid-IR preparation of either *j* = 1.5 *e* or *f*, a very low-intensity residual spectrum is obtained, shown (multiplied by a factor of 30) by the black inverted line. Comparison of this spectrum and the *j* = 1.5 *e*/*f* prepared spectra shows that all the residual population arises without mid-IR excitation, and hence there is no evidence for intrabeam collisions transferring population between the *j* = 1.5 *e* and *f* levels after mid-IR preparation.

We believe that the residual signals are the result of the *v* = 1 population in the gas reservoir behind the pulsed valve (partition ratio ≈1 × 10^–4^ at a room temperature of 293 K) being “frozen out” in the supersonic expansion. The vibrational relaxation of NO­(*v* = 1) by Ar via vibration-translation (V-T) coupling is known to be a very inefficient process, with a measured room temperature rate constant *k*_V‑T_(Ar) ≈1 × 10^–17^ cm^3^ s^–1^.^57^ V-T and vibration–rotation (V-R) relaxation of NO­(*v* = 1) by NO­(*v* = 0) is about 10^4^ times faster.^58^ In a typical molecular beam, the average number of collisions in the expansion is expected to be of order of 10^2^ – 10^3^,^59^ while *k*_V‑T_(Ar) implies >10^6^ collisions are required for relaxation. In our dilute 0.1% NO/Ar beam, the NO self-relaxation rate will be comparable to that from Ar, and hence overall we do not expect significant NO­(*v* = 1) relaxation in the expansion. The observed rotational population distribution is also consistent with that of the NO­(*v* = 0) in our NO/Ar molecular beams, further supporting this hypothesis. Following this assumption, the ≈500-fold enhancement in *j* = 1.5 signal with mid-IR excitation is consistent with an optical pumping efficiency of ≈5%. The residual population in *j* = 0.5 *F*_1_ *e*/*f* is ≈100 times smaller than that of the prepared *j* = 1.5 *F*_1_ *e*/*f*. Overall, the prepared state purity in *v* = 1 is ≈99%, comparable to the state purity obtained in NO­(X) *v* = 0 using hexapole state selection.^1^ We observe the same rotational state distribution in the *j* = 0.5 *e*/*f* and the unprepared *j* = 1.5 Λ-doublet for both the residual NO and state-prepared scans, indicating that collisional redistribution by intrabeam collisions following state preparation is also negligible in our experiments.

Clearly, with the observed frequency stability of the QCL, we can effectively select either the *e* or *f* Λ-doublet. However, for the *f* Λ-doublet, where the hyperfine transitions are fully resolved, this is at the expense of efficient signal acquisition as the QCL slowly drifts on and off the transition over the typical 1–2 h time scale of a scattering experiment. This is why we chose to prepare the *e* Λ-doublet for the scattering experiments, as the closely spaced hyperfine levels result in consistent rotational and parity-selected state preparation. We also tested whether the initial *j* = 1.5 *F*_1_ *e* state had a prepared rotational angular momentum alignment by switching the linear polarization of the probe laser to be either parallel or perpendicular to the QCL polarization while detecting the prepared level. No measurable intensity difference was observed, consistent with no prepared alignment. Although the action spectra are poorly resolved for the *e* Λ-doublet, we are unlikely to be exciting multiple hyperfine levels for any individual probe laser pulse. Hence, we would not expect to observe the effective nuclear hyperfine depolarization that would arise from coherent excitation of all of the hyperfine levels with a broadband laser pulse.^60^ An alternative explanation for the observed lack of alignment may lie in depolarization from stray magnetic fields in the ≈10 μs flight time from preparation to probe region. A prepared alignment will precess around an applied magnetic field axis that is not parallel to the quantization axis, resulting in a time-dependent oscillation of the alignment at twice the Larmor frequency, ω_*L*_.^61^ Inhomogeneous fields can hence cause effective depolarization of a prepared alignment on a time scale that depends on ω_*L*_, which itself depends on the gyromagnetic ratio for the relevant rotational state and the magnetic field strength. For the *j* = 1.5 *F*_1_ *e* initial state, a magnetic field of 50 μT, similar to the strength of Earth’s magnetic field in Edinburgh, results in a Larmor precession frequency ω_*L*_ ≈ 1 × 10^5^ rad s^–1^.^62^ The experimental apparatus is not shielded against magnetic fields, and it therefore seems reasonable to assume that stray inhomogeneous fields of similar strength to Earth’s field may be present and hence be responsible for the observed depolarization.

In Figure 2 we present Newton diagrams for the NO + Ar and NO + CH_4_ systems, superimposed on the experimental images, summed over the V and H geometries. In both cases, the initial prepared state is *j* = 1.5 *F*_1_ *e*, and the final state is *j′* = 10.5 *F*_1_ *f*. The velocity vectors reflect the mean of the respective measured molecular beam speeds, with an outer circle representing the in-plane scattered speed of the NO final state. Additional rings are drawn on the diagram for CH_4_, representing the in-plane speeds of NO *j′* = 10.5 *F*_1_ *f* formed in coincidence with varying energy transfer to the unobserved CH_4_, specifically (outermost to innermost) $\Delta E_{CH_{4}}$ = 0, 60, 120, 180, 240, and 300 cm^–1^. The Newton diagram for Ar overlaps very well with the sharp outer ring observed in the data. The sharp edge to the scattering signal reflects the good collision-energy resolution and low ion recoil and imaging blurring, combined with the energy and momentum conservation constraints imposed by true state-to-state scattering with a monatomic collider. The image drops to very low intensity across its center, reflecting the projection involved in crushing a thin shell in velocity space onto the 2-dimensional detector.^63^ The propagation direction of the probe laser, ***k***_**p**_, is nearly perpendicular to the relative collision velocity, ***k***. This results in the electric vector of the probe laser, ***ε***_**p**_, lying nearly parallel to ***k*** for the H-geometry. As a result, the experiment is only sensitive to product rotational angular momentum moments with rank *k* = 2, and projection *q* = 0 and *q* = 2+, for these collisions.^3,30,31^

**Figure 2:**
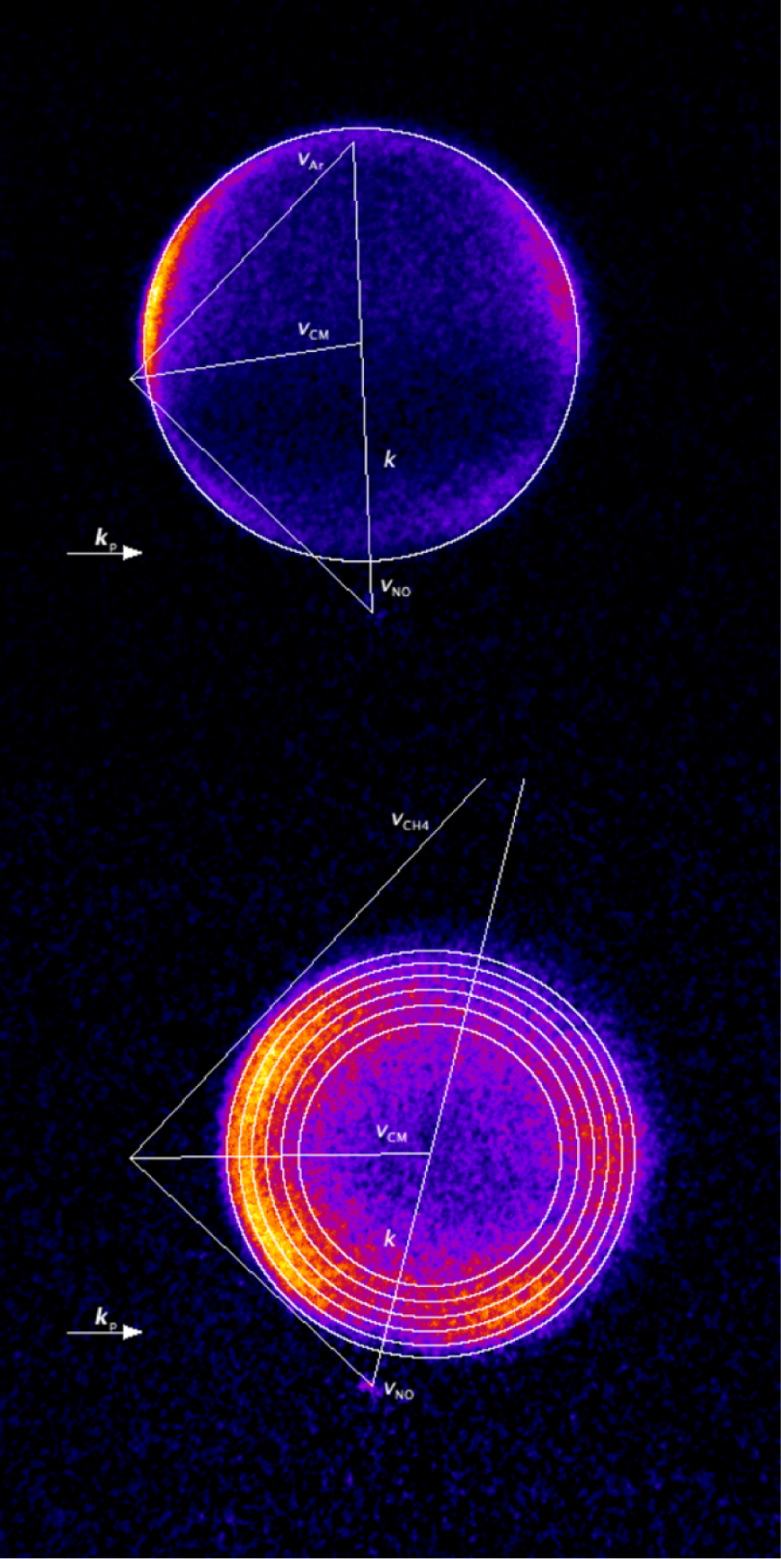
Newton diagrams for scattering from initial state *v* = 1, *j* = 1.5 *F*_1_ *e* to final state *v* = 1, *j′* = 10.5 *F*_1_ *f* for collision with Ar (top) and CH_4_ (bottom), superimposed on summed V + H geometry experimental data images. The four vectors are the laboratory frame velocities of NO (***v*** **_NO_**), Ar or CH_4_ (***v*** **_Ar_** or **v_CH4_**, respectively), the velocity of the center of mass (***v*** **_CM_**), and the relative collision velocity (***k***). The rings represent the in-plane scattering velocities for the average collision energies of the two systems, with the multiple rings shown for CH_4_ representing transfer of energy to the (unobserved) rotational states of CH_4_, respectively 0, 60, 120, 180, 240, and 300 cm^–1^. The arrow labeled ***k*** **_p_** represents the propagation direction of the resonant probe laser beam, relevant for the relative sensitivities of the V and H geometry images to the product angular momentum polarization moments.

In contrast, the high speed of the CH_4_ beam rotates ***k*** in the laboratory frame, and ***ε***_**p**_ no longer lies parallel to ***k*** in the H-geometry. The experiment is therefore also sensitive to the angular momentum moment *k* = 2, *q* = 1+, for the NO + CH_4_ collisions.^3,30–32^ It is also immediately obvious that this image has a much broader outer intensity ring and that the center has much higher intensity than the NO + Ar image. Although some of this is a consequence of the higher spread of speeds in the CH_4_ beam, and hence a larger spread of collision energy, as we will show in the fits below, the primary origin of this is energy transfer to rotation of the CH_4_.

We first fitted the NO + Ar scattering images, which provide a stringent test of our determination of the experimental parameters and our modeling of the resulting images. We used a development of our existing fitting code, which has been described in detail previously.^30–32,34^ Briefly, Monte Carlo sampling from the known initial experimental conditions, including molecular beam spatial overlap, molecular beam velocity distributions, and ion-recoil and image blurring, is used to simulate basis images representing discrete DCS basis functions, in this case defined by Legendre polynomials in cosθ, where θ is the polar differential scattering angle. In this work, we have extended this Monte Carlo procedure to include flux-density effects that were not significant in our earlier published work on NO­(A^2^Σ^+^), but which do need to be considered here. A linear combination of these basis images is least-squares fitted to the experimental images, and the resulting weighting coefficients are used with the DCS basis functions to recover the overall DCS. An extension of this forward simulation and back-fitting procedure has been previously used by us to also extract scattering angle-dependent angular momentum tensor moments. However, we expect the angular momentum alignment in the NO­(X) + Ar system to be dominated by hard-shell interactions, as previously reported by Brouard and coworkers for NO­(X, *v* = 0) + Ar.^3,45^ We have therefore instead included the effect of the $A_{0}^{\left(2\right)}\left(\theta \right)$ , $A_{1+}^{\left(2\right)}\left(\theta \right)$ and $A_{2+}^{\left(2\right)}\left(\theta \right)$ alignment moments predicted by the QS calculations in the basis images for the V and H-geometry images, and have then fitted these polarization-dependent basis images simultaneously to the two geometries.^30^ Trial fitting revealed that while the angle dependence of the alignment moments was well reproduced, the overall magnitude of the effects was smaller in the experimental data than expected. We believe that this is the result of partial saturation in the A-X­(0,1) probe step, a consequence of the relatively high fluence used in the experiments. Reducing the predicted sensitivity for all alignment moments to 70% of their literature value resulted in excellent agreement between experiment and QS predictions, and all subsequent fitting used this reduced sensitivity.

Figure 3 shows the experimental data, and the resulting fits, for both final states, *j′* = 4.5 *F*_1_ *f* and *j′* = 10.5 *F*_1_ *f*, in both V and H geometries, as well as the subtraction image, V – H. For each final state, the fit procedure was independently performed on 5 separate measurements of the pair of V and H images, and the resulting data and fit images were coadded for presentation purposes. From inspection of the V and H images, it is immediately obvious that the agreement between the fit and data is excellent for both final states. Very good agreement is also observed in the V – H subtraction images, confirming that the QS calculations provide a very good representation of the rotational angular momentum alignment. Scattering into *j′* = 4.5 has a large peak around θ = 0°, with low but varying intensity around the sideways and backward directions. The V–H difference image is positive (V-geometry larger) at the forward peak and displays weak negative intensity sideways and backward. This represents a positive $A_{0}^{\left(2\right)}\left(\theta \right)$ over the forward region, transforming into negative $A_{0}^{\left(2\right)}\left(\theta \right)$ through the sideways and backward scattering angles, and is clearly predicted by the QS calculations. The DCS for final state *j′* = 10.5 is substantially different, with little or no scattering around θ = 0°, and two peaks centered around 45° and 120°, respectively. The peak of the DCS moving to a higher scattering angle with increasing rotational excitation is a well-known general pattern in rotationally inelastic scattering, with its origin in the requirement for collisions with lower impact parameters to generate the larger linear-to-angular momentum transfer associated with transfer to higher *j′*. The V–H image is predominantly negative across the scattering angles with substantial population, consistent with a negative $A_{0}^{\left(2\right)}\left(\theta \right)$ , again predicted by the QS calculations.

**Figure 3:**
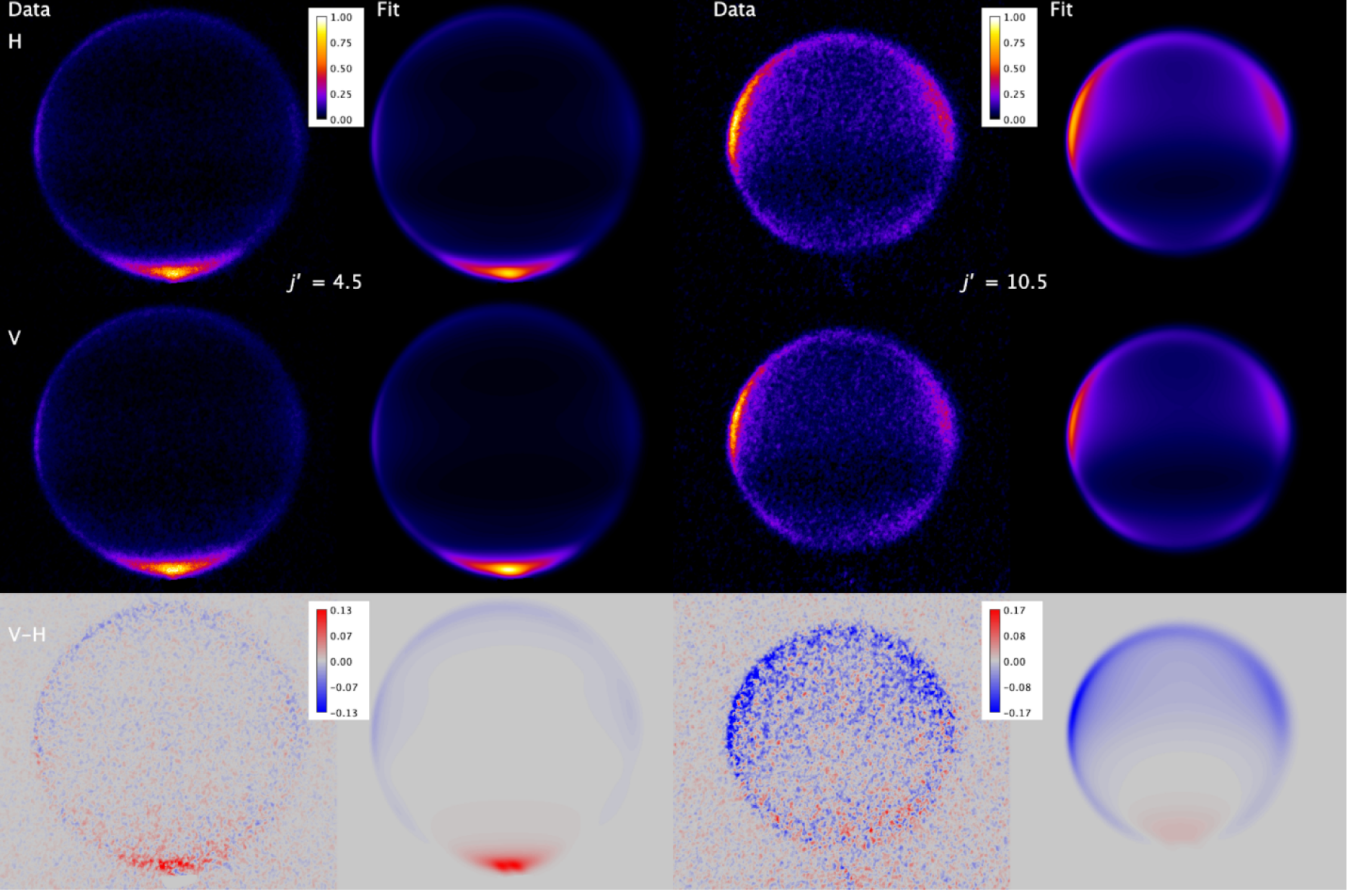
Experimental data and fits for NO­(X, *v* = 1, *j* = 1.5 *F*_1_ *e*) + Ar to final states *j′* = 4.5 *F*_1_ *f* (data column 1 and fit column 2) and *j′* = 10.5 *F*_1_ *f* (data column 3 and fit column 4). Rows in sequence are H-geometry, V-geometry, and V–H. The 5 individual measurements made for each final state have been independently fitted and then added for presentation here.

Figure 4 shows the DCSs resulting from the fits to the two final states and compares them to the QS predictions. The experimental DCS has been normalized to the integral cross-section predicted by the QS calculations to enable comparison. For *j′* = 4.5, the QS calculations show high-frequency oscillations in the DCS in the 0–20° region with a period of ≈2°. The experimental angular resolution, determined from Monte Carlo simulation, is ≈5° in this region, and we therefore do not resolve these oscillations. At larger angles, from 30° to 180°, we observe low-amplitude scattering with a sequence of maxima and minima, the locations of which are well reproduced by the QS calculations. Overall, we observe slightly lower amplitude forward scattering and correspondingly slightly higher backward scattering than is predicted by theory.

**Figure 4:**
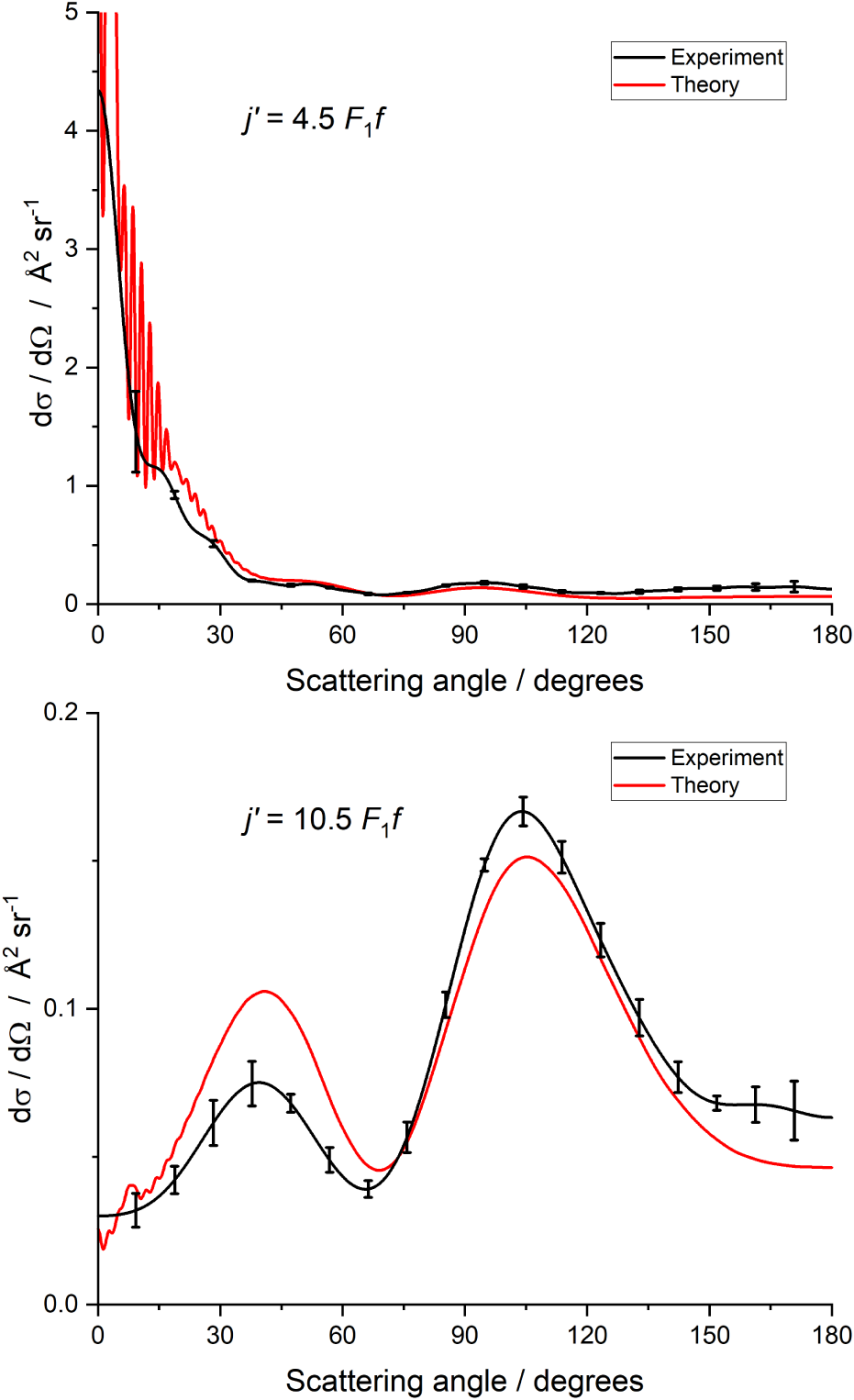
Differential cross sections for NO­(X, *v* = 1, *j* = 1.5 *F*_1_ *e*) + Ar to final states *j′* = 4.5 *F*_1_ *f* (top panel) and *j* = 10.5 *F*_1_ *f* (lower panel). Experimentally determined DCS (black line, error bars are 1 standard error of the mean) and collision-energy-averaged quantum scattering calculations (red line).

For *j′* = 10.5, both the experimental and QS DCSs display two clear maxima at 40° and 104°, respectively. There is excellent agreement in the location of these scattering peaks, but also a clear disagreement in their relative intensity, with the experiment once again displaying lower relative intensity in the forward hemisphere.

The excellent agreement between experiment and theory for the NO + Ar scattering gives us confidence that we understand the experimental parameters. We have therefore fitted the NO + CH_4_ scattering images using the “peeling” approach that we have previously used to analyze NO­(A) + N_2_, O_2_, CO, and CO_2_ scattering, again with modification of the Monte Carlo sampling to introduce appropriate density-flux corrections.^35,36^ In brief, this fitting approach considers discrete user-defined energy transfers to the unobserved partner, $\Delta E_{CH_{4}}$ , sequentially fitting and removing contributions from the experimental images. First, the software simulates basis images using the same Monte Carlo sampling procedure as the NO + Ar fitting, assuming that zero collision energy is transferred to the unobserved partner. These basis images are least-squares fitted to the outermost region of pixels that contribute to this energy transfer, ignoring the inner regions of the image, which would be expected to have significant contributions from scattering with higher $\Delta E_{CH_{4}}$ . The resulting DCS for this initial $\Delta E_{CH_{4}}$ is then used to simulate complete V and H images, which are subtracted from the data images to remove the contribution of this energy transfer to all pixels; this is the “peeling” step. The process is then repeated for the rest of the user-defined $\Delta E_{CH_{4}}$ , determining DCSs in sequence and removing their contributions from the data, until the final set of basis functions is fitted to the entire remaining image. The experimental H and V geometry images showed significant intensity differences as a function of scattering angle, indicating that relatively strong angular momentum polarization was present in the scattering. No QS predictions of the angular momentum polarization are available for the NO­(X) + CH_4_ system. We therefore included the effects of the $A_{0}^{\left(2\right)}\left(\theta \right)$ , $A_{1+}^{\left(2\right)}\left(\theta \right)$ and $A_{2+}^{\left(2\right)}\left(\theta \right)$ alignment moments, predicted by KA conservation for each of the $\Delta E_{CH_{4}}$ , here 0, 60, 120, 180, 240, and 300 cm^–1^, on the V and H basis images.

In Figure 5, we present experimental and fitted images for both final state *j′* = 4.5 *F*_1_ *f* and 10.5 *F*_1_ *f*, for both H and V geometries, and the V–H subtraction for NO + CH_4_. On first inspection, the experimental images have (surprising) similarities to the NO + Ar images. Scattering into *j′* = 4.5 peaks sharply around θ = 0°, but also extends at low intensity across the sideways and backward directions, as observed for the same state with Ar. Similarly, for *j′* = 10.5, the scattering displays a double peak split on either side of θ = 90° with lower intensity forward and backward. However, in both cases, the images show a much broader ring than that observed for scattering from Ar. As noted previously, although the collision energy spread is larger for NO + CH_4_ than for NO + Ar, it is not large enough to explain the observed images. We demonstrate this in SI Section S.3 by fitting the NO + CH_4_ images with the nonpeeling approach applied to the NO + Ar images, which proves to be a comprehensively inadequate model for NO + CH_4_. This is clear evidence for energy transfer to the rotation of CH_4_, quantitatively determined by the “peeling” fitting procedure. The fit images agree very well with the experimental images for both final states and both H and V geometries. This is reflected in the V–H subtraction images, where the generally very good agreement indicates that KA conservation of the rotational angular momentum polarization is a reliable model for these NO­(X) + CH_4_ collisions.

**Figure 5:**
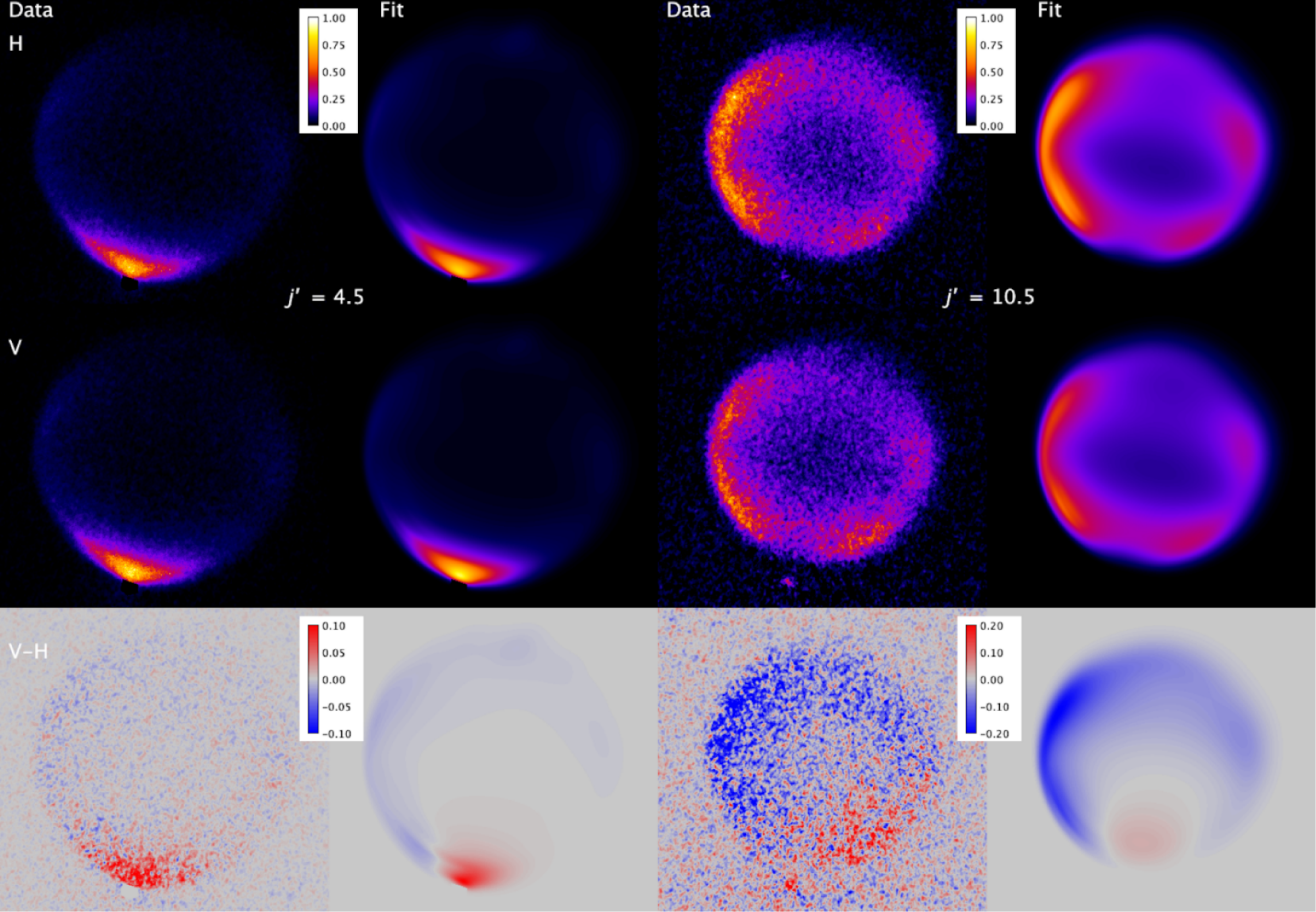
Experimental data and fits for NO­(X, *v* = 1, *j* = 1.5 *F*_1_ *e*) + CH_4_ to final states *j′* = 4.5 *F*_1_ *f* (data column 1 and fit column 2) and *j′* = 10.5 *F*_1_ *f* (data column 3 and fit column 4). Rows in sequence are H-geometry, V-geometry, and V–H. The 8 and 9 individual measurements made for *j′* = 4.5 and 10.5, respectively, have been independently fitted and then added for presentation here.

Overall, the quality of the fits to the data gives us confidence in the $\Delta E_{CH_{4}}$ energy-dependent DCSs derived from them. These are shown in Figure 6, where the upper panel is for final state *j′* = 4.5, and the lower panel is for *j′* = 10.5. In each case, individual DCSs are shown for the six defined $\Delta E_{CH_{4}}$ , along with the total DCS formed from the sum of the individuals. For *j′* = 4.5, as expected from the images, the total DCS is strongly forward-scattered, with small peaks sideways and backward. The individual DCSs are consistent with relatively strong transfer of energy into CH_4_ rotation, in comparison to the 35 cm^–1^ that has been transferred into the NO. In all the individual DCSs, the maximum is at θ = 0°, with the sideways peak at θ = 100° arising from the 0 cm^–1^ DCS. Between θ = 20° and θ = 45°, the second and third DCSs ( $\Delta E_{CH_{4}}$ = 60 and 120 cm^–1^ respectively) are larger than the $\Delta E_{CH_{4}}$ = 0 cm^–1^ DCS.

**Figure 6:**
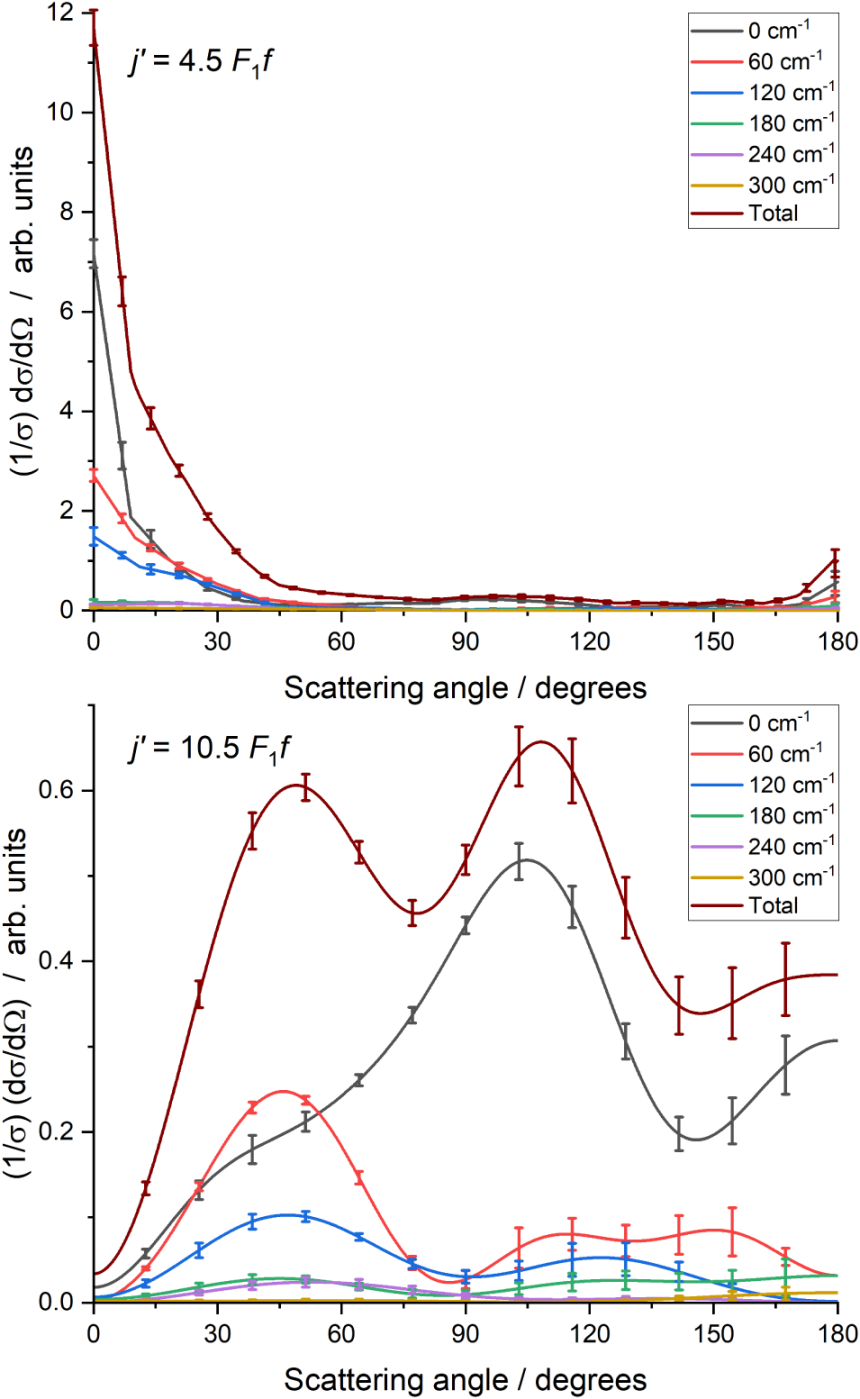
Differential cross sections for NO + CH_4_ scattering, as a function of energy transferred to rotation of the CH_4_, for final states *j′* = 4.5 *F*_1_ *f* (top panel) and *j′* = 10.5 *F*_1_ *f* (lower panel). Lines are the mean of 8 (*j′* = 4.5) and 9 (*j′* = 10.5) separate fits to independent measurements, error bars are 1 standard error of the mean. The total DCS is the sum of the contributions from each individual DCS, and the error bars are 1 standard error.

The total DCS for *j′* = 10.5 is also consistent with the images, with maxima at θ = 50° and θ = 110°. The individual DCSs also vary significantly as a function of $\Delta E_{CH_{4}}$ , with the second maximum at θ = 110° dominated by the $\Delta E_{CH_{4}}$ = 0 cm^–1^ DCS, and the first maximum having its largest contribution from $\Delta E_{CH_{4}}$ = 60 cm^–1^, with another substantial contribution from $\Delta E_{CH_{4}}$ = 120 cm^–1^.

Finally, Figure 7 shows the integral cross sections for both *j′* = 4.5 and 10.5 as a function of $\Delta E_{CH_{4}}$ , determined by integration of the individual DCSs and normalized to unity total cross section. Only ≈40% of the total cross section for *j′* = 4.5 is the result of scattering with $\Delta E_{CH_{4}}$ = 0 cm^–1^, with a nearly monotonic decline in cross section across the remaining $\Delta E_{CH_{4}}$ . Surprisingly, for *j′* = 10.5, a larger fraction, ≈63%, of the total cross section is provided by $\Delta E_{CH_{4}}$ = 0 cm^–1^. After a large drop to σ_tot_ ≈ 0.2 for $\Delta E_{CH_{4}}$ = 60 cm^–1^, the cross sections then also decline monotonically. This anticorrelation of the rotational energy transfer (higher $\Delta E_{CH_{4}}$ for lower Δ*E*_NO_) is not a consequence of conservation of total energy, as even for $\Delta E_{CH_{4}}$ = 300 cm^–1^ and Δ*E*_NO_ = 194 cm^–1^ (*j* = 10.5), there is still >200 cm^–1^ of collision energy available. The average energy transferred to CH_4_ for each state may be calculated from these cross sections, yielding $\langle \Delta E_{CH_{4}}\rangle =67\pm 3$ cm^–1^ for *j′* = 4.5 and $\langle \Delta E_{CH_{4}}\rangle =38\pm 5$ cm^–1^ for *j′* = 10.5 (2 sigma errors).

**Figure 7:**
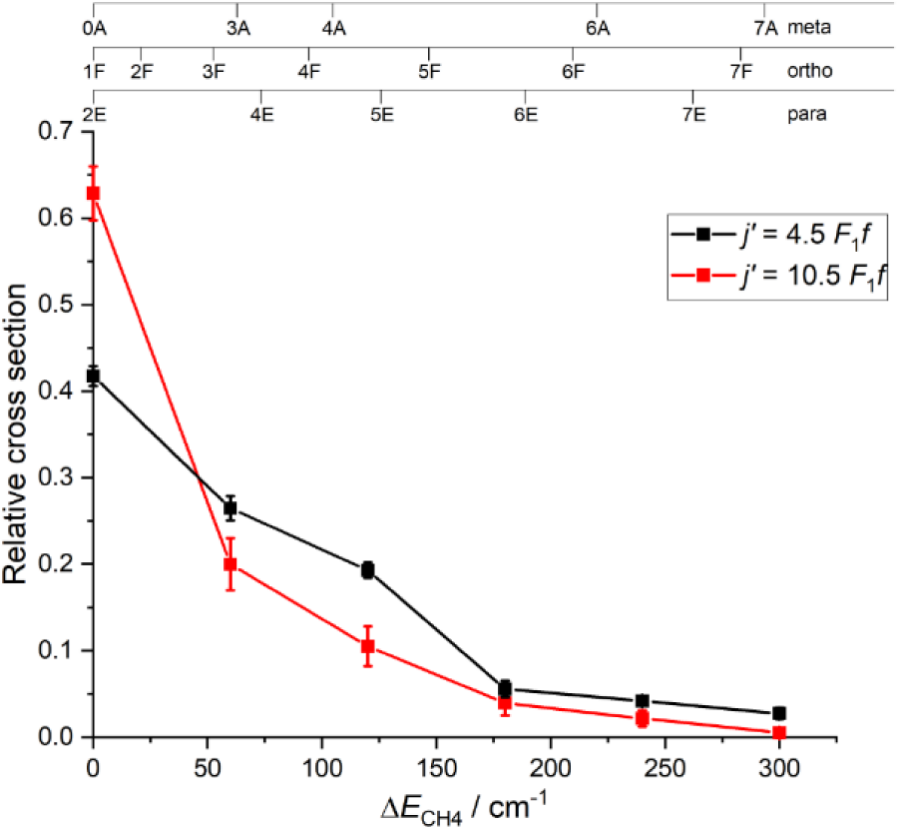
Integral cross sections as a function of energy transferred to CH_4_, summed to unity overall. Final states *j′* = 4.5 *F*_1_ *f* (black symbols and error bars) and *j′* = 10.5 *F*_1_ *f* (red symbols and error bars). The symbols are the mean of fits to the 8 (*j′* = 4.5) or 9 (*j′* = 10.5) independent measurements, and the error bars are 2 standard errors of the mean. The lines are included purely to guide the eye.

## Discussion

We first discuss the scattering in collisions with Ar. As noted in the introduction, this collision system is among the most intensively studied of all historically, with a very wide range of experimental and theoretical reports of state-resolved DCSs. The majority of these are, as expected, for scattering of NO­(X, *v* = 0), with a smaller number of recent studies of highly vibrationally excited NO­(X), in both *v* = 9 and *v* = 10.^2,3,7,20,21,23–26,39,40,43–45,64^ However, to the best of our knowledge, there are no previous reports of initial-to-final rotational and parity state-selected DCSs for *v* = 1 with Ar, providing the opportunity to explore the consequences of excitation of a single quantum of vibrational energy. Turning first to final state *j* = 10.5 *F*_1_ *f*, we observe two clear rainbow peaks, whose location, but not relative intensity, are accurately predicted by the QS calculations. Similar multipeaked DCSs have been widely observed in inelastic scattering of NO­(X) with rare gases for state-to-state transitions that conserve rotational parity.^1,4,5^ The origins of this structure have been extensively investigated via both experiment and theory by Brouard and coworkers, using the NO­(X, *v* = 0) + Ar system as a test case.^2,20,21^ They conclude that the multiple peaks are a consequence of interference between different pathways that lead to the same final rotational state, specifically between collisions that sample the two different ends and the sides of the NO. Because parity-conserving and parity-changing collisions have different sensitivities to the even and odd components of the PES, the parity-changing final states typically have a single rainbow peak. These interference effects are therefore largely obscured in experiments which, in contrast to those presented here, do not initially prepare the NO­(X) in a single parity-defined state.^65^ A further consequence of the parity dependence of inelastic scattering is the observation of *parity pairs*, where final rotational states of opposite parity differing in *j′* by one have almost the same DCS.^19^

The comprehensive experiments by Brouard and coworkers on NO­(X, *v* = 0) + Ar were performed at the same collision energy as the experiments presented here, and their accompanying QS calculations also used the same PES.^20^ The initial rotational state they prepared was *j* = 0.5 *F*_1_ *f*. The final state in our measurements, *j′* = 10.5 *F*_1_ *f*, is the result of a *Δj* = +9 parity-changing collision. Comparing equivalent *changes* in rotational angular momentum, we therefore consider their final state *j′* = 9.5 *F*_1_ *e* and its parity-pair state *j′* = 10.5 *F*_1_ *f*. The experimental DCSs reported by Brouard and coworkers for this parity pair are, within experimental error, very similar to the DCS reported here for *j′* = 10.5 *F*_1_ *f*. Brouard and coworkers also report experiment-theory agreement that differs in the relative size, but not location, of the two scattering peaks, and is comparable to our experiment-theory agreement. This suggests that the introduction of one quantum of vibrational excitation has no measurable effect on the DCSs and that QS calculations using a fixed *r*_*e*_ NO bond length and associated PES are accurate enough to reproduce the rotational rainbow scattering. A recent scattering experiment by Kamasah et al. used a flash-heated molecular beam source to generate a rotationally cold NO (*v* = 1) sample, enabling direct comparison of *v* = 0 and *v* = 1 rotationally inelastic DCSs in scattering with Ar, from an ≈ 20 K initial rotational state distribution.^66^ Within experimental error, they observed the same DCSs for scattering to specific final *j′* for both *v* = 0 and *v* = 1, as well as excellent agreement with QS calculations on the Alexander PES, consistent with our conclusions.

The scattering-angle-dependent angular momentum alignment observed in our experiments is very well described by the QS calculations and is also very similar to the *v* = 0 experiment and theory reported by Brouard and coworkers. As stated in the introduction, these alignment moments can also be predicted with high accuracy by conservation of the initial rotational angular momentum in the KA frame.^3,45^ This is a hallmark of collisions dominated by hard-shell interactions, which is consistent with the known potential and the repulsive-wall-mediated rotational-rainbow scattering observed in the final state *j′* = 10.5.

The most striking feature of the DCS for scattering to *j′* = 4.5 *F*_1_ *f* is the high-frequency oscillations in the 0–20° region, which are not resolved by the experiment. This is another common feature of NO­(X) inelastic scattering with rare gases and is also a consequence of interference effects, in this case, diffraction of long-range collisions around the “hard shell” repulsive wall of the NO­(X)-Ar potential. We note that it is the limits of the experimental angular resolution, arising both from collimation of the molecular beams and the longitudinal spread of beam speeds, that prevent us from resolving these oscillations; they have not been washed out by the spread of collision energies, which have been included in the QS predictions. Van de Meerakker and coworkers have explored the diffraction oscillations in NO­(X) + rare gas collisions in great detail across a range of colliders, using the very high *angular* resolution provided by their Stark decelerator preparation of the NO­(X) to resolve peaks separated by only 1–2°.^6,7,64^ These high-frequency oscillations are also present in the rotational alignment moments predicted by our QS calculations, although they are of course also washed out in the images by the angular resolution of our experiment. The overall envelope of the product polarization moments, within this diffractive oscillation, is again very similar to a prediction from the KA conservation model, something that again has previously been observed for *v* = 0 scattering.^3,45^ Hard-shell scattering therefore dominates even for this low-*j′* final state, either through diffraction at small scattering angles or through rainbow scattering across higher angles.

The low-intensity wide-angle scattering features we observe for *j′* = 4.5 are hence from repulsive-wall interactions and are again in good agreement with scattering to the equivalent final states in the *v* = 0 measurements.^21^ The only significant disagreement between experiment and QS predictions is the relative intensity of forward and backward scattering for both final states. We considered the possibility that the experiments were systematically biased toward detecting backward scattering. However, this seems unlikely given the experimental arrangement of the molecular beams and the probe and ionization beams, which ensure that forward and backward scattered products are essentially equally likely to be probed before loss from the probe volume. In contrast, the intensity dependence on azimuthal angle, which we expect to have a strong dependence on final laboratory-frame speed through flux-density effects, is well-modeled by our Monte Carlo analysis code. We therefore believe that the similar level of agreement with theory for *j′* = 10.5 in both the literature *v* = 0 and new *v* = 1 experiments indicates small inaccuracies in the PES used in both cases.^21,55^

Turning to the results of scattering from CH_4_, we first consider *j′* = 4.5. The total DCS is very similar to that observed for scattering from Ar, with a large peak centered around θ = 0°, and low-intensity side and backward scattering. However, there is significant energy transfer to CH_4_ rotation in this dominant forward-scattered peak. Forward scattering with energy transfer to the collision partner has been observed before in both NO­(X) and NO­(A) scattering. In our own experiments on NO­(A) + CO_2_ inelastic scattering, we observed forward scattering correlated with significant rotational excitation of the CO_2_, which we attributed to long-range glory scattering on a very strongly attractive and anisotropic PES.^36^ Van de Meerakker and coworkers have shown that the forward scattering with partner rotational excitation that they have observed in the collisions of NO­(X) with CO, O_2_ and HD can be explained by the effect of attractive forces acting after the initial rotational excitation in a low-impact-parameter collision, a general inelastic scattering mechanism that they have named hard-collision glory scattering (HCGS).^12,13,29^ However, neither of these mechanisms are likely to be relevant in the NO­(X) + CH_4_ scattering presented here, because the potential well depth is too small relative to the experimental collision energy. RCCSD­(T) calculations of the NO­(X)–CH_4_ PES, which are in good agreement with infrared spectroscopy of the van der Waals complex, give a potential minimum *V*_min_ = 177 cm^–1^ for a *C*_*s*_ symmetry arrangement of the NO relative to the CH_3_ face.^67–69^ The forward scattering that is associated with $\Delta E_{CH_{4}}$ = 0 cm^–1^ may be similar to that observed in the NO­(X) + Ar scattering, and hence be dominated by long-range diffractive scattering. However, the components associated with modest energy transfer to CH_4_ ( $\Delta E_{CH_{4}}$ = 60 and 120 cm^–1^) are scattered into a wider range of forward hemisphere angles, implying they arise from stronger, largely repulsive interactions. Further evidence for this is supplied by the observed angular momentum polarization, which is consistent with KA conservation, also an indication of repulsive-wall interactions.^1,19,45^

The total DCS for scattering into *j′* = 10.5 is also very similar to that observed for Ar scattering, and the strong scattering into sideways and backward directions with NO rotational excitation is a classic indicator of repulsive-wall interactions. However, as with *j*′ = 4.5, there are clear correlations of the DCS with $\Delta E_{CH_{4}}$ , which make direct comparisons with Ar scattering dangerous. The interference effects that lead to the double-peaked total DCS in NO­(X) + Ar scattering cannot be the cause of the double-peaked DCS observed here, as the two peaks correspond to two different scattering channels with varying $\Delta E_{CH_{4}}$ . The forward hemisphere peak at θ ≈ 45° is predominantly from scattering with $\Delta E_{CH_{4}}$ > 0 cm^–1^, suggesting a repulsive-wall interaction, which is again supported by the excellent agreement of the KA polarization predictions with experiment. This does not mean that interference effects are not present in the specific energy-correlated channels, and they may, for example, be the origin of the double-peaked structures in the higher $\Delta E_{CH_{4}}$ channels. However, extension of the 4-path model used by Brouard and coworkers to explain the interference effects in NO­(X) + Ar to the higher-dimensionality NO­(X) + CH_4_ system would be challenging, notwithstanding the absence of an appropriate hard-shell potential, and we have not attempted any such modeling.^2^

The overall observation of significant energy transfer to CH_4_ with a negative correlation to NO final state *j′* is in broad agreement with the only previous study of NO­(X) + CH_4_ rotationally inelastic scattering by Orr-Ewing and coworkers.^47^ It is surprising because only positive rotation–rotation correlations have been reported in all the other NO­(X) and NO­(A) molecule–molecule scattering systems studied.^11–14,16,29,33,35,36^ With the exception of the recent work on NO­(X) + ND_3_, in all cases, the collision partner was a linear molecule, and with only one further exception, NO­(A) + CO_2_, the collision partner was a diatomic. This raises the possibility that it is the change of shape of the molecule from linear to tetrahedral that has provided a new scattering pathway, responsible for the negative correlation. Orr-Ewing and coworkers did not resolve the DCS as a function of $\Delta E_{CH_{4}}$ , and based solely on the negative correlation they observed for overall energy transfer (*j*′ - $\Delta E_{CH_{4}}$ ) proposed a simple model. In their model, high-*j′* NO will, as in collisions with other collision partners, be the result of lower impact parameter interactions, which are required to provide large torques on the NO. However, because CH_4_ has its center of mass at the molecular center, a low impact parameter collision will provide it with only a small torque, and hence lower rotational excitation. They suggested that, in contrast, high impact parameter collisions would generally produce low rotational excitation of NO, as observed with atomic collision partners, but would in contrast provide a significant torque on the CH_4_, leading to high CH_4_ rotational excitation. This is, of course, a simple model constructed in the absence of any support from *ab initio* theory on the hyperdimensional shape of the NO–CH_4_ PES.

Somewhat surprisingly, however, our higher-resolution, fully state-selected experiments do provide some additional information that is, if not in outright agreement, also not clearly in disagreement with this model. Not only do we see the same overall negative *j′–* $\Delta E_{CH_{4}}$ correlation, but the DCSs for both *j′* = 4.5 and 10.5 show forward hemisphere repulsive wall scattering that correlates with increased rotation in CH_4_, as qualitatively predicted for the larger impact parameter channel in the above model. Similarly, the backward hemisphere peak observed for *j′* = 10.5 is predominantly correlated with lower rotational excitation in CH_4_, which would be qualitatively predicted by the lower impact parameter collision pathway in the model.

These two product states therefore give us a tantalizing glimpse of the dynamical correlations underlying molecule–molecule RET with larger molecules. Our resolution of the DCSs as a function of $\Delta E_{CH_{4}}$ , which was not possible in the experiments reported by Orr-Ewing and coworkers, has been enabled by the preparation of a single initial NO quantum state. Clearly, a more extensive exploration of the range of product states produced in NO­(X) + CH_4_ scattering, for example, spin–orbit and/or parity-changing collisions, would give a wider evidence base for modeling the collision dynamics, including the importance and influence of multiple-path interference effects in molecule–molecule scattering. However, a fundamental limit on the resolution of coincident rotational excitation comes from the spread of collision energies. A possible route to improve the collision energy spread, without the application of a Stark or Zeeman decelerator, would be to use the narrow line width of a QCL counter-propagating the molecular beam to Doppler select a subset of the molecular beam velocity distribution, as recently demonstrated by Krohn and Chandler.^70^ In addition, scattering calculations are an invaluable companion to experiment, and we hope that high-resolution experimental results such as those presented here will encourage both calculations of the complete PES and the development of associated scattering calculations, e.g., quasi-classical scattering, quantum scattering, or, given the relative complexity of the system, extending recent developments in mixed quantum-classical scattering theory.^71^

## Conclusions

We have used a mid-IR DFB-QCL to prepare a single rotational and parity-selected state of NO­(X, *v* = 1), specifically *j* = 1.5 *F*_1_ *e*, in a CMB-VMI apparatus and have studied state-to-state rotationally inelastic scattering of NO­(X, *v* = 1) with both Ar and CH_4_ to two representative final states, namely *j′* = 4.5 *F*_1_ *f* and *j′* = 10.5 *F*_1_ *f*. In scattering from Ar, the DCSs and NO rotational angular momentum alignment for both final states are in excellent agreement with QS predictions on a literature *ab initio* PES calculated with NO fixed to its equilibrium bond length, implying that the relatively small change in average NO bond length arising from excitation to *v* = 1 has no measurable effect on rotationally inelastic scattering dynamics. Significant transfer of collision energy to rotational excitation of CH_4_ is observed for both final states, with larger overall CH_4_ rotational energy for NO final state *j′* = 4.5, in broad agreement with earlier experiments performed without full initial state selection on NO­(*v* = 0) scattering from CH_4_.^47^ For NO *j′* = 10.5, the coincident CH_4_ rotational excitation is correlated with scattering angle, with higher CH_4_ rotational excitation for scattering into the forward than the backward hemisphere. This contrasts with the positive rotation–rotation correlations observed in NO­(X) scattering from diatomic molecules such as CO and O_2_, providing a glimpse of the varied and complex dynamical correlations underlying molecule–molecule scattering that remain to be explored.^11–14^

## Supplementary Material



## Notes

Ion optics design: simulations of the electric fields and ion trajectories; computer-aided design renders of the mechanical design; integration of the ion optics into the experimental apparatus (Section S1). Ion optics calibration: example ion images from O_2_ photodissociation as a function of repeller voltage for both crush and dc-slicing; speed-pixel calibration as a function of repeller voltage; speed-pixel calibration using NO_2_ photodissociation at +600 V repeller voltage (Section S2). NO + CH_4_ fitting: result of fitting NO + CH_4_ images with the CH_4_ acting as a pseudoatom (Section S3) (PDF)
