# Supplementary material for: Optical Selection of Rotational and Parity-Resolved States for Rotationally Inelastic Scattering: NO(X2Π1/2, v = 1, j = 1.5e) with Ar and CH4
Source: J Phys Chem A. 2026 Mar 9;130(11):2362–75. doi: 10.1021/acs.jpca.5c08389 (PMC13007015; doi:10.1021/acs.jpca.5c08389)
Supplement: Supplementary file 1 [file jp5c08389_si_001.pdf]

Optical Selection of Rotational and Parity-Resolved States for Rotationally Inelastic Scattering: $NO(X^2\Pi_{1/2}, v = 1, j = 1.5e)$ with Ar and CH_4

Supporting Information

Martin Fournier, Rebecca G. Cameron, Kenneth G. McKendrick and Matthew L. Costen

Institute of Chemical Sciences, School of Engineering and Physical Sciences, Heriot-Watt University, Edinburgh EH14 4AS, United Kingdom

1. Ion Optics Design

The position, shape, and the applied voltages for the ion optics were designed using SIMION (version 8.1.1.32-2013-05-20, Scientific Instrument Services, Inc.) simulation of the electric fields and sample ion trajectories. The mechanical design was then completed in a computer-aided design (CAD) package (Autodesk Inventor Professional 2023) to provide a complete three-dimensional model of the ion optics and their mounting elements, which could be placed within an overall model of the experimental apparatus. Finally, the ion optics and mounting elements

were manufactured in-house at Heriot-Watt, with the electrodes machined from phosphor bronze, mechanical support rods from polyether ether ketone (PEEK, 6 mm diameter) and insulating spacers, also PEEK (6 mm inner diameter ID, 10 mm outer diameter, OD). The OD (80 mm) of the ion optics was defined by their clearance once completed and including electrical cabling, though the largest aperture on the experimental scattering chamber, a CF100 flange. The overall flight distance from the ionization region to the detector surface plane was (arbitrarily) set to 1000 mm. The general design principle behind the ion optics was that of Tkac *et al.*,[1] itself based on an original time-slicing ion optics design proposed by Lin *et al.*[2] This design principle separates the optics into several separate regions. The ion optics are cylindrically symmetric in the *x*-*y* plane, with the time-of-flight (TOF) along the *z*-axis. The first region contains the initial ion formation and acceleration. Here the fields are as flat as possible in the *x*-*y* plane and the electric field gradient is kept low to allow TOF spread for ions formed from neutrals with different initial *z*-axis velocities. The ions exit this region and pass through a focusing region, provided by electrodes 4 through 8. The third region provides the majority of the overall ion acceleration, and here the electric fields are again flat in the *x*-*y* plane, finishing with a grounded electrode.

The main section of the ion-optics assembly was constructed from 4 different types of electrodes: the repeller, standard, stabilization, and ground. Figures S1 to S4 show CAD models of these individual electrode types.

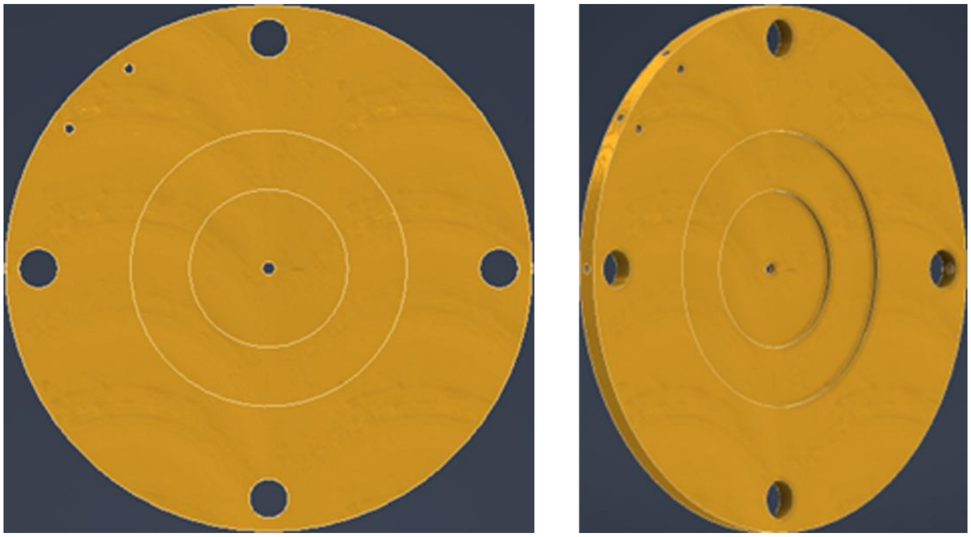

Figure S1. CAD render of the repeller plate front (top) view and ¾ view.

The repeller electrode, shown in Figure S1, was solid, except for a 2 mm diameter central aperture, which allowed molecular beam access along the TOF *z*-axis for photodissociation-based calibration experiments. It was composed of a 3 mm thick outer disk, 80 mm OD, with a 2 mm thick, 42 mm OD middle disk, and 1 mm thick, 24 mm OD, inner disk. Four 6 mm diameter apertures centered at a radius of 35 mm provided clearance for the support rods. Additional 1.5 mm diameter apertures provided venting for these supports. Finally, interconnected edge and plate apertures 1.5 mm in diameter provided access for electrical connection.

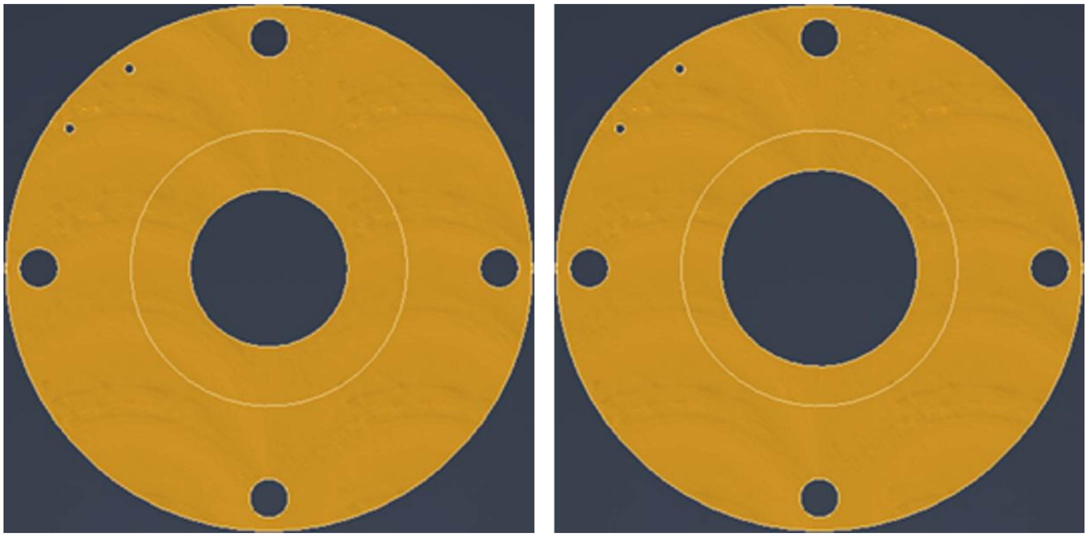

Figure S2. Control electrode (left, used for elements 2, 3, 4 and 8) and the standard electrode (right, used for elements 5, 6, 7 and 9 to 20), top view.

The control electrode and the standard electrode were of similar design, shown in Figure S2, comprised of three circular regions. The outer region was a 3 mm thick disk of 80 mm OD, within that was a 1 mm thick region of 42 mm OD, with a central aperture of either 24 mm or 30 mm diameter, respectively. The final, grounding electrode, shown in Figure S3, was constructed as an extended hyperboloid cone, opening from an ID of 30 mm at the input to a final exit ID of 48 mm. All these electrodes have the same support post and electrical connecting apertures as the repeller electrode. This ground electrode was electrically connected to the chamber via a shorting plug on the appropriate electrical feedthrough, and the chamber was then grounded externally.

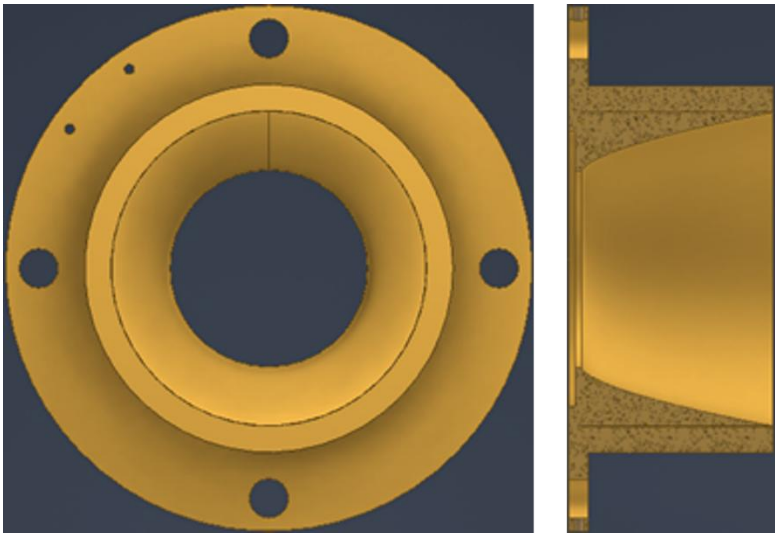

Figure S3. Top and side view renders of the ground electrode with OD 80 mm. The inner opening has been replaced by a hyperboloid defined by the parametric curve $x(t) = 1.6t^2 + 2; y(t) = 1.09t + 15$ where t $[0, 8.2]$ is the grid unit of the model.

Most of the electrodes were evenly spaced by 7 mm (center to center), with the exceptions of the electrodes in the extraction region. Here the spacing of electrodes 1 to 2 and 2 to 3 (surrounding the experimental interaction region) was 9 mm, allowing space for molecular beam and laser access and providing the most uniform fields in this critical region, while electrodes 3 and 4 were 8 mm apart. The voltages on the repeller (electrode 1) and extractor (electrode 4), along with control electrodes 2 and 3, were independently provided by a precision high voltage power supply (ISEG GmbH, EHS 80 60p, 8 channel, max. voltage +6000 V, < 10 mV ripple). These 4 electrodes formed the repeller-extractor field. Electrodes 4 to 8 were connected by an in-vacuum resistor chain, with Electrode 8, and hence the fields in the focusing region between electrodes 4 and 8, directly controlled by the same HV power supply. Electrodes 8 to 21 (the ground electrode) were also connected by an in-vacuum resistor chain. The resistors had a

resistance of 219.3 ± 0.1 Ω, having been individually selected and tested from a large batch with nominal 220 Ω resistance. With +1000 V applied to the repeller plate, the independent voltages applied to electrodes 2, 3, 4 and 8 were +960 V, +915 V, +875 V and +706.25 V, respectively. The field gradients were 5 V / mm between electrodes 1 and 4, nominally 6.03 V / mm in the lens region between electrodes 4 and 8, and 7.76 V / mm in the acceleration region between electrode 8 and the ground electrode.

Crossed molecular beam scattering results in an image that is centered on the velocity of the center of mass of the collision system in the laboratory, and the scattering image is hence generally not mapped to the center of the detector. We therefore included a deflector in the VMI design to allow us to steer the position of the complete image on the detector, shown in Figure S4. The deflector is composed of 16 individually addressable plates that can generate a field orthogonal to the propagation axis, allowing the deflection of the complete ion cloud in any direction on the MCP-Phosphor detector. The plates were cut from a cylinder of phosphor bronze with OD of 44 mm and ID of 40 mm and are 40 mm in length. They are mounted on a grounded phosphor bronze cylinder of 48 mm ID, from which they are electrically isolated by a Teflon® sleeve of OD 48 mm and ID 44 mm. The outer cylinder is electrically grounded to the same point as the ground electrode. The deflector is controlled by a 16 channel 1000 V power supply (ISEG GmbH EHS F5 10p, 10 mV ripple), with each deflector plate independently addressed. The deflector is mounted immediately after the ground electrode, providing the full field-free TOF path for deflection. To steer the image, a voltage is applied to the electrodes, generating a potential gradient in the desired direction. By pairing electrodes on opposite sides of the cylinder we generate a locally flat field, using the following equation to determine the voltage applied to each of the deflector electrodes, which are spaced by 22.5°.

$$V_n = M\big(1 + cos(\theta - \theta_n)\big) \quad (S1)$$

Here V_n is the applied voltage on electrode at angle θ_n, M is the desired magnitude of deflection, and θ is the desired deflection angle from an (arbitrarily selected) laboratory direction parallel with one axis of the camera image. At a total ion-optic potential of +600 V, a magnitude M = +10 V, which applies V_n varying between 0 and +20 V across the deflector, moves the center of the image 17 mm across the MCP detector.

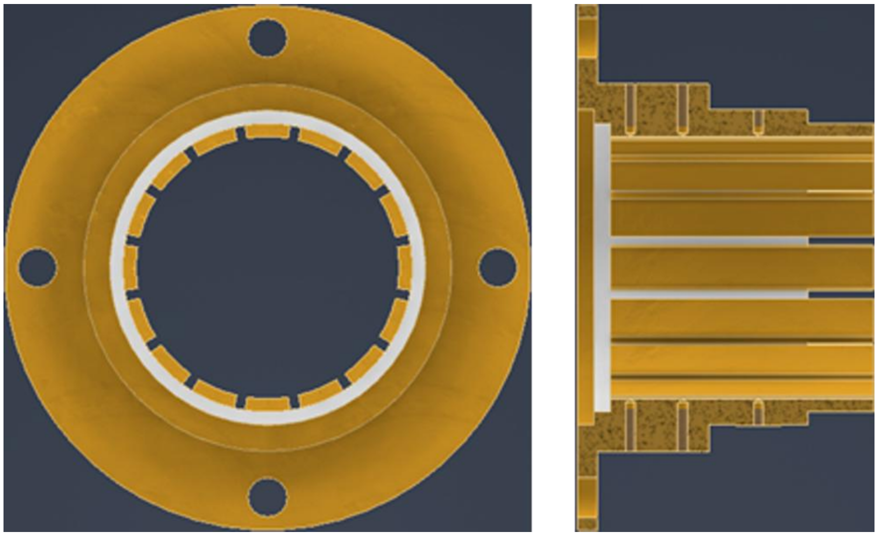

Figure S4. A render of the deflector design. Head-on view (left panel), side-on view (right panel). The white part is made from Teflon® to insulate the outer shell, which is electrically grounded, from the deflection plates.

Figure S5 shows a complete CAD render of the entire ion optics assembly as a cut-away, including showing the mounting of the deflector relative to the main ion-optics stack.

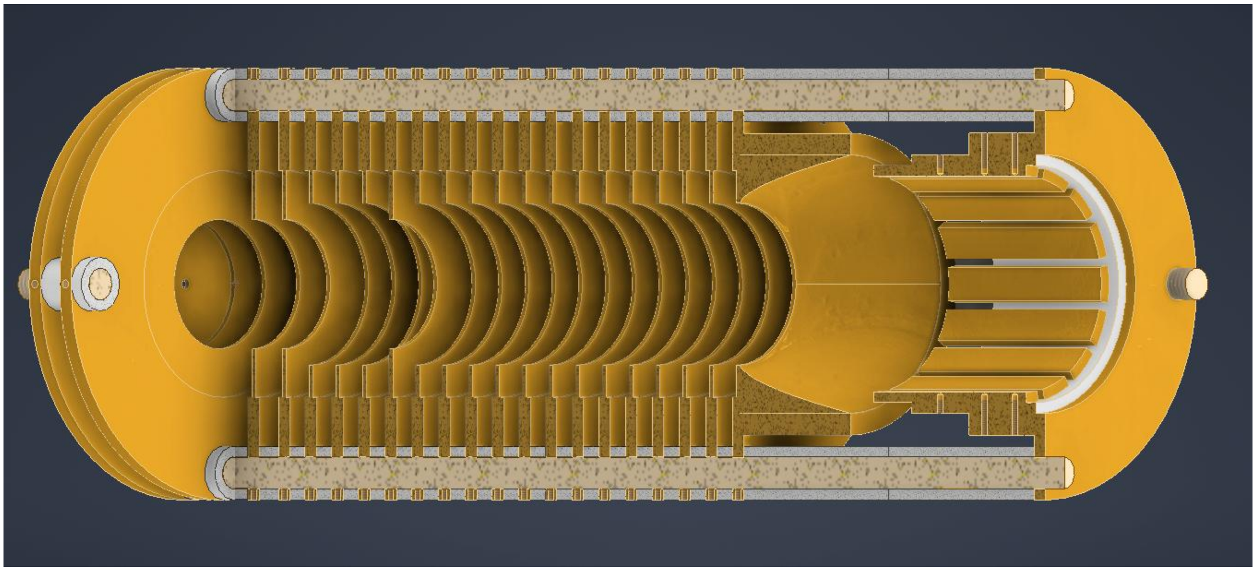

Figure S5. A CAD render of the assembled VMI detector; ¾ view using a cut along the vertical plane and the laser-molecular beam plane. Electrodes 1 and 2, and those with a smaller ID (3, 4 and 8) are directly controlled by the HV power supply, the remainder are linked by in-vacuum resistors.

Figure S6 shows a SIMION simulation for the final ion optics design, with a repeller electrode potential of +600 V. The upper panel shows the complete ion trajectory through the ion optics and the field-free region to the detector plane. The lower panel shows an expanded view of the electrode assembly and the resulting electric fields with 15 V contours. Also shown are a selection of ion trajectories for two different neutral / initial ion kinetic energies in the *x*-*y* plane and mass-to-charge ratio m/z = 30. Ions with both kinetic energies are focused to points on the detector plane with radial positions proportional to the square root of the kinetic energy and a

size comparable to the diameter and spacing of individual channels on a typical microchannel plate (10 μm).

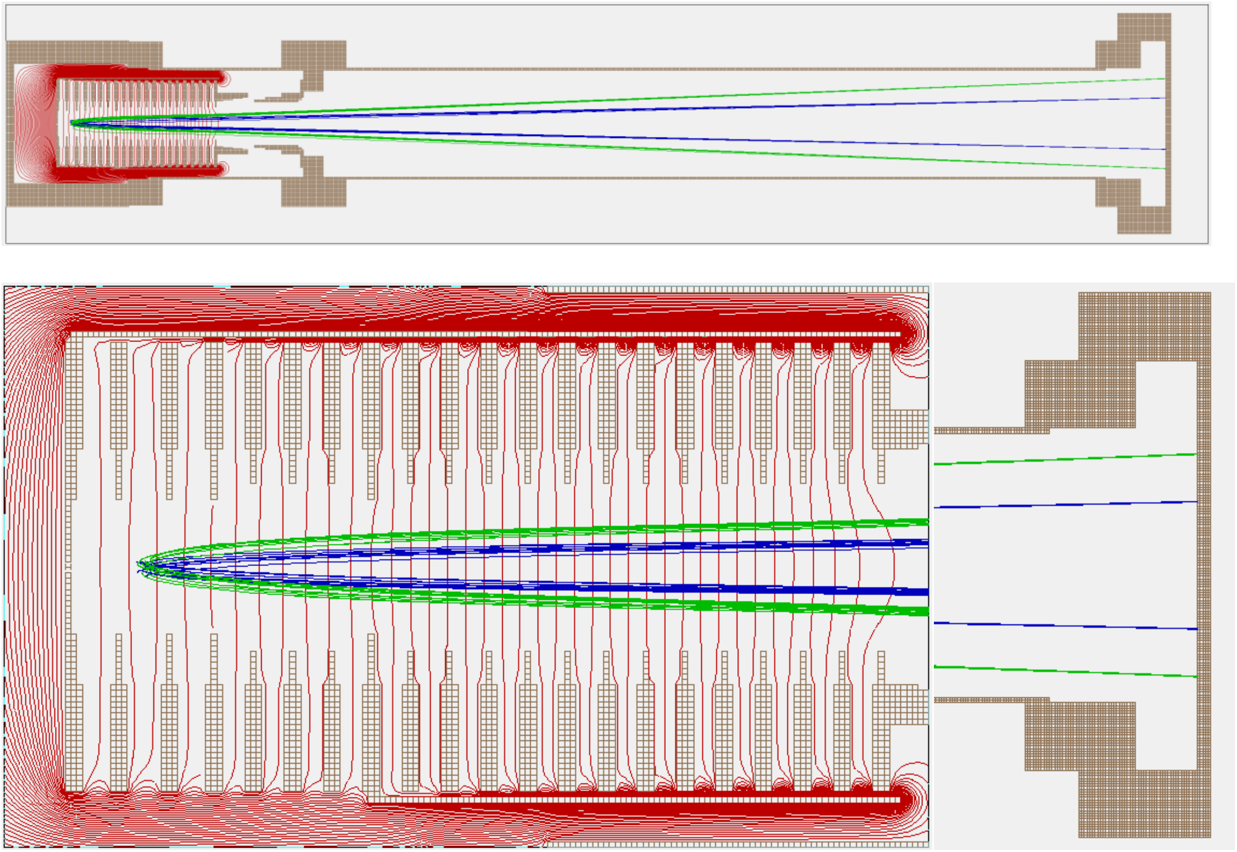

Figure S6. Top panel, SIMION simulation of the entire imaging assembly, lower panel expanded view of the SIMION simulation and particle trajectories with a cut on the x-y plane removing the field-free flight tube. The simulation grid size is 1 mm. Electric field contours (red lines) at 15 V intervals from 0 to + 600 V. Trajectories for ions with m/z = 30, a start position randomly selected from a Gaussian distribution (σ = 1 mm) and an initial velocity along either +y or -y. Kinetic energy = 0.136 eV (blue lines) or 0.5 eV (green lines).

Finally, Figure S7 shows a CAD render of the completed ion optics assembly mounted in the VMI apparatus. The support rods for the ion optics and deflector are fixed to the lower surface of a CF100 custom double-sided flange, which is placed between the TOF tube and a custom 6-way cross (2 x CF100, 4 x CF40, 149 mm between the CF100 flanges), which itself sits on top of the existing scattering chamber. The 4 CF40 flanges provide access for the electrical connections to both the ion optics and the deflector. Not shown in this figure are the flight tube and detector chamber. These were also upgraded from our previously published work. The flight tube is 't-shaped', 432 mm long with CF100 end flanges, and is evacuated through a CF63 side port by a turbomolecular pump (Edwards nEXT 85H). A CF100 gate valve (Highvac 11110-0404) on the top of the TOF tube separates the scattering chamber from the detector chamber, allowing independent venting. The detector chamber is formed from a standard 4-way reducing cross, with 2 CF100 flanges and 2 CF63 flanges. One CF63 flange is used for a turbomolecular pump (Edwards EXT 76DX) and the other for an ion gauge (Edwards AIGX-S). The detector is mounted on a CF160 flange and is coupled to the detector chamber by a zero-length CF100-CF160 adaptor flange.

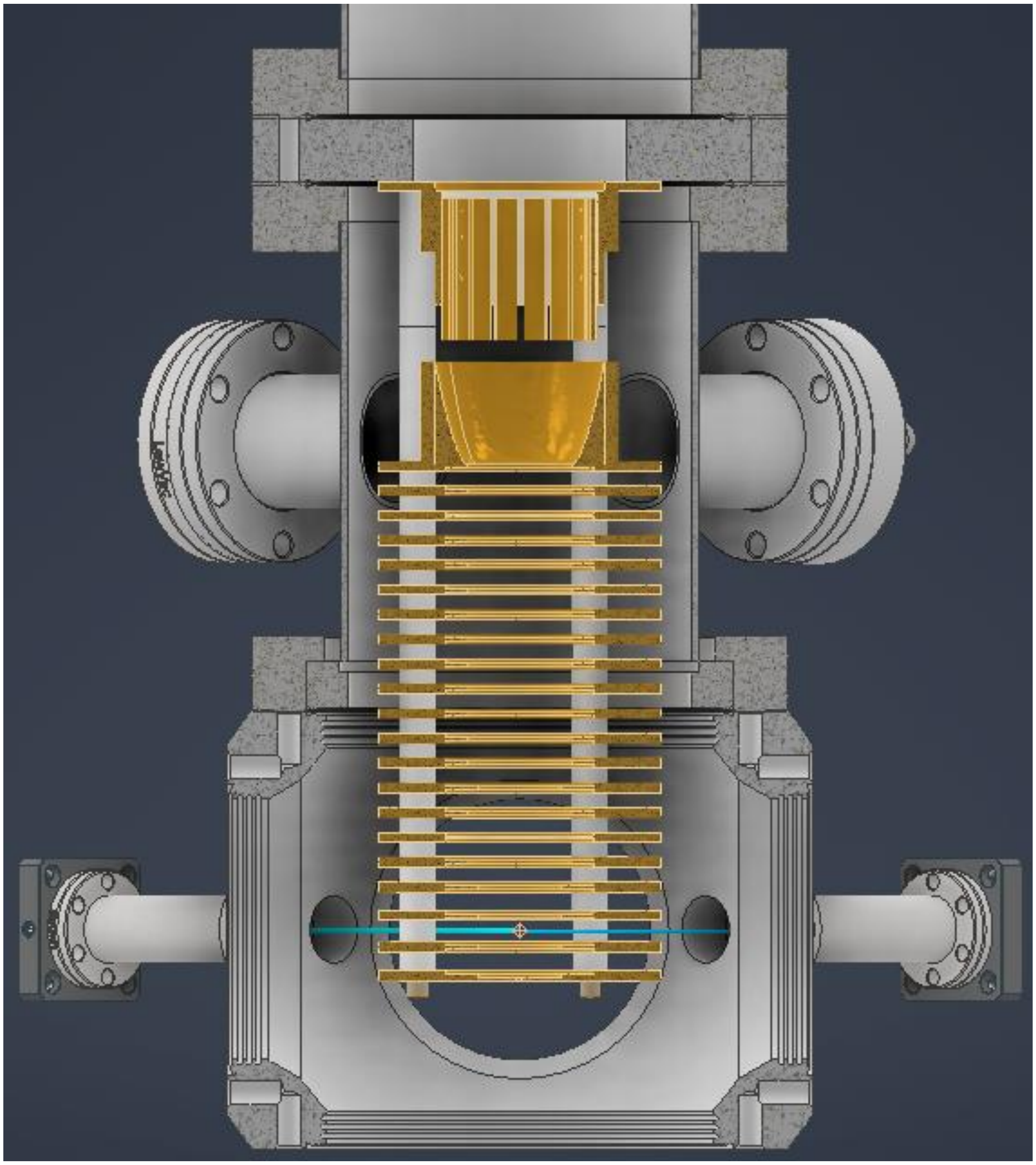

Figure S7. Cutaway view of the ion optics installed in the scattering chamber and the custom length cross DN100-DN40 6-way cross. The ground electrode and the deflector main body of the ion optics stack are grounded to the chamber. The blue and cyan lines indicate the probe and ionization laser paths, defining the center of the VMI region. The position and diameter of the posts was optimized to leave clearance relative to all 8 ports of the main scattering chamber for laser and molecular beam access.

2. Ion Optics Calibration

Two different photodissociation-based procedures were used to test and calibrate the ion optics design. The first was the widely used mixed photodissociation/photoionization of O_2 with one-color excitation at 224.999 nm, for which the energetics of all of the major channels have been previously determined.[3-4] A skimmed molecular beam of pure O_2 (BOC, 99.999%) was directed through the 2 mm aperture in the repeller electrode and intersected by a focused 224.999 nm laser pulse (250 mm focal length lens, typically 100 μJ). Ions were detected at a TOF consistent with m/z = 16 in both crush and dc-slice imaging modes. Figure S8 shows both crush (left hand side) and dc-slice (right) images for an applied repeller voltage of +4000 V, suitable for high kinetic energy fragments. At this high ion optics potential, the TOF is short and hence the TOF spread of the O^+ packet is relatively short. For crush imaging the detector was therefore gated with a 200 ns HV pulse, while for the dc-slice imaging a 30 ns HV pulse was applied. Note that the effective gating time of the detector is shorter than the applied pulse length, and pulses shorter than ≈ 17 ns resulted in either spatially uneven imaging or no increase in MCP gain and hence no visible ion strikes. The minimum gate time that can be reliably applied is therefore 20 ns. The excellent quality of the images for both crush and dc-slicing, with high velocity resolution and a high degree of cylindrical symmetry confirm that the ion optics meet their design specification.

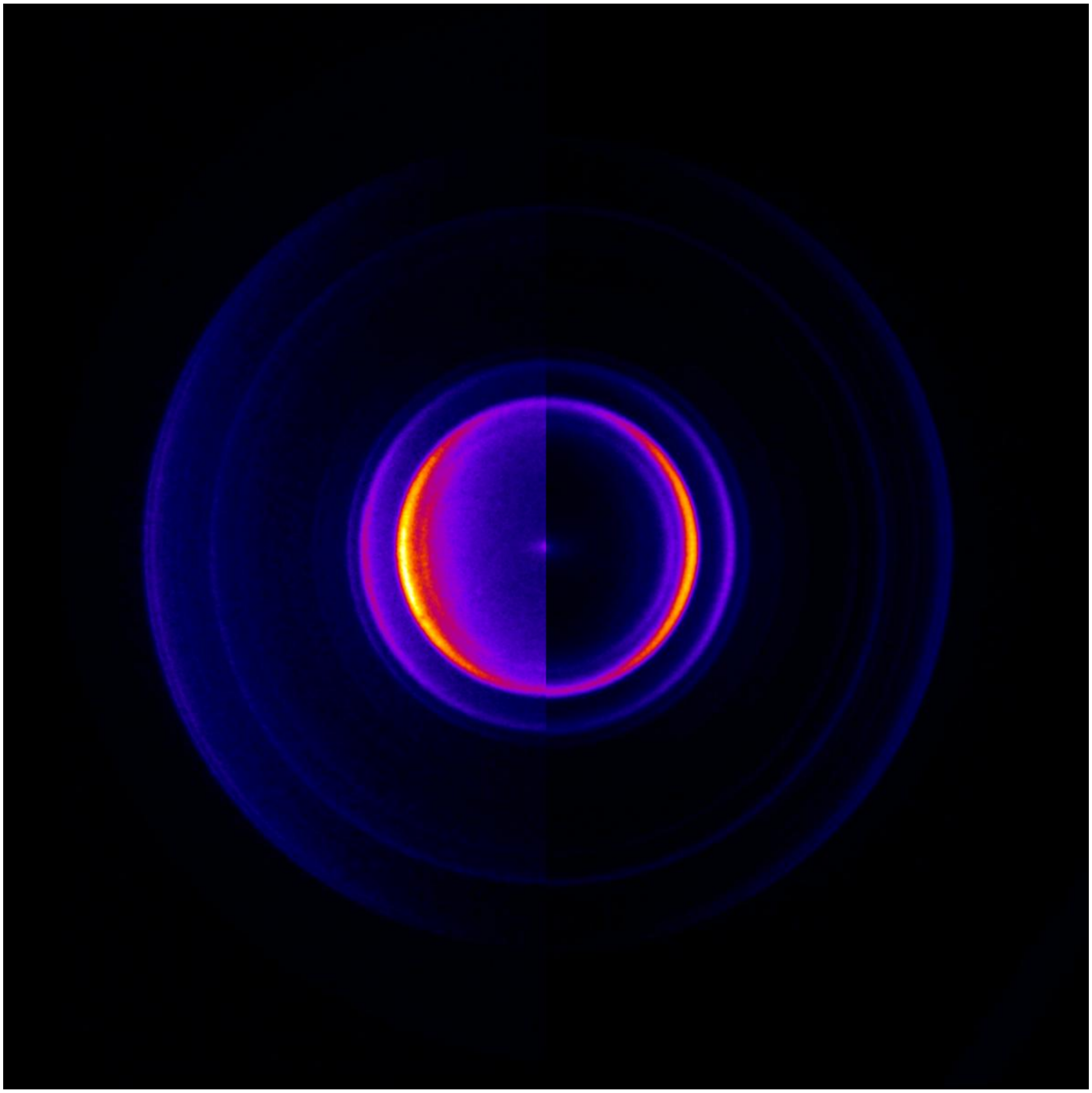

Figure S8. Images from photodissociation of O_2 at 224.999 nm with +4000 V applied to the repeller electrode. The left half of the image was acquired under crush conditions (200 ns MCP gate width), and the right side of the image was acquired under dc-slicing conditions (30 ns MCP gate width). The total voltage across the MCPs was +1300 V, increasing to +1800 V during the gate, with +4000 V on the phosphor screen.

Figure S9 is a composite of quadrants from 4 separate dc-sliced ion images of O^+ from O_2 photodissociation / photoionization at different repeller electrode voltages, +4000 V, +2000 V, +1500 V and +1000 V, respectively, showing that the imaging conditions are maintained as the overall potential across the ion optics is reduced.

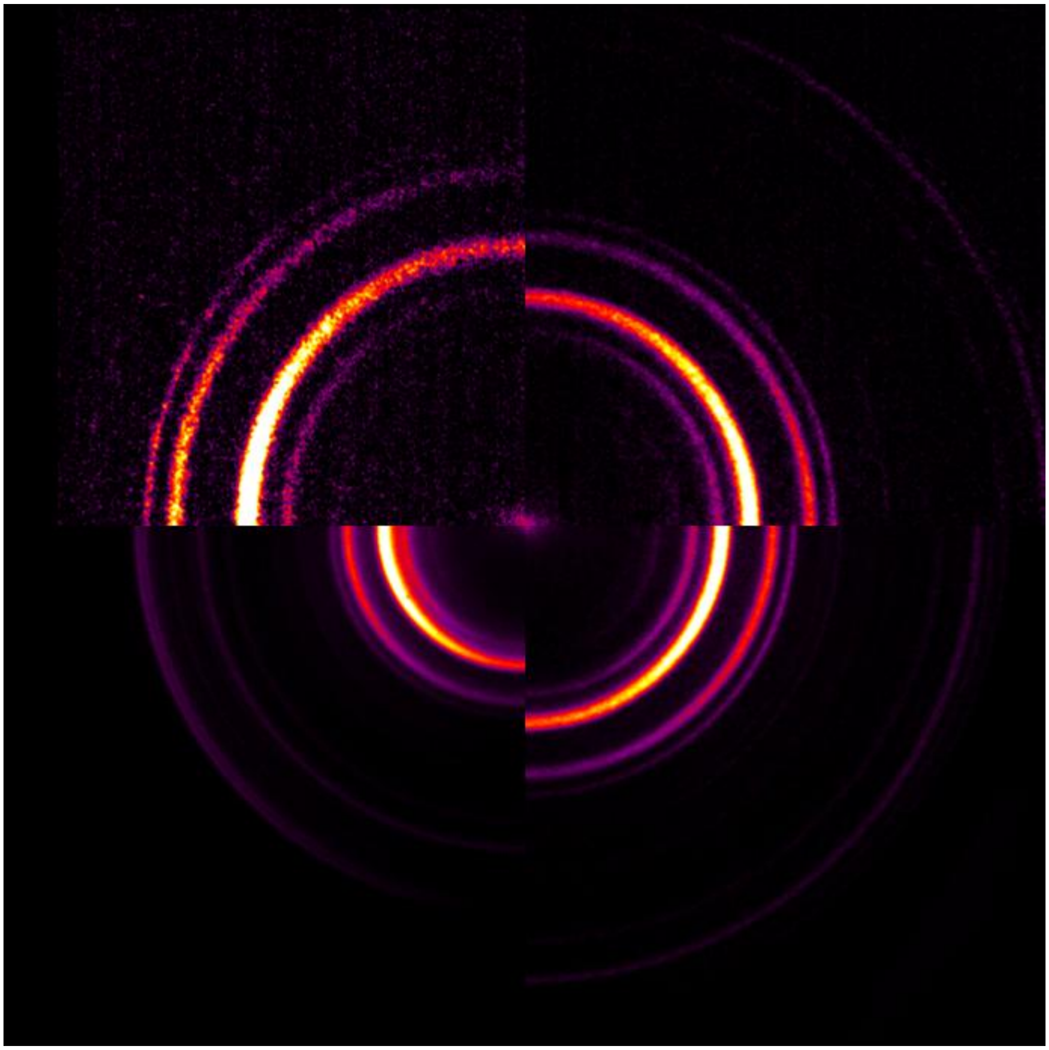

Figure S9. Photodissociation image of O_2 at 224.999 nm and +1000 V repeller (top left), +1500 V (top right), +2000 V (bottom right), +4000 V (bottom left). Images obtained using dc-slicing with a 30 ns gate width, over 5000 laser shots.

Each of the images was analyzed using literature information on the kinetic energy release for specific channels to determine the speed-to-pixel ratio as a function of the overall potential across the ion optics. The calibration factor is dependent on the mass-to-charge of the detected species, through the dependence on the flight time on this quantity. We have therefore corrected the O^+ calibration by $\sqrt{16/30}$ to yield the appropriate calibration factor for NO^+. The calibration factors for ion-optic potentials ranging from +1000 V to +4800 V determined in this fashion are shown in Figure S10.

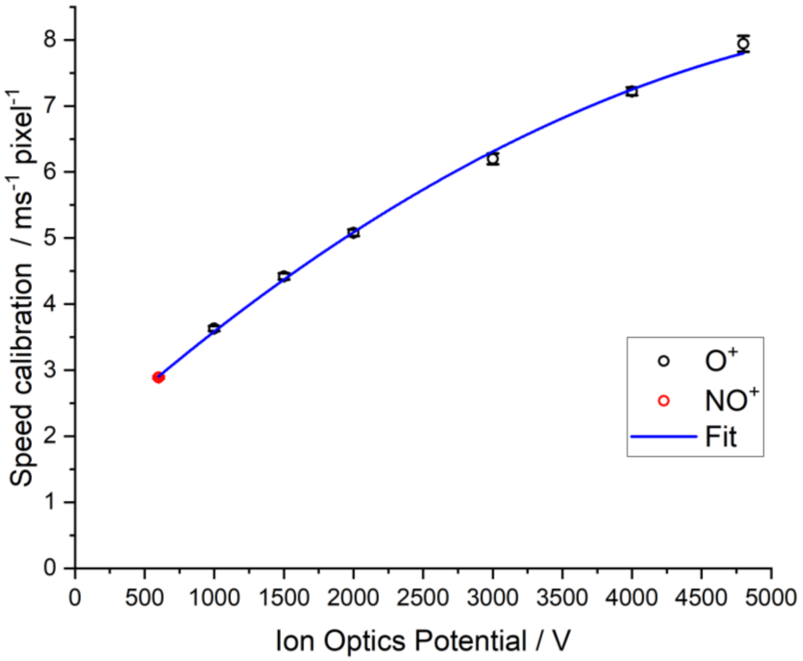

Figure S10. Speed-to-pixel calibration factor for *m*/*z* = 30 as a function of the potential across the ion optics. Black open circles from O_2 photodissociation, red open circle from NO_2 photodissociation, with 2σ standard errors. Solid blue line is a 2nd order polynomial fit.

The O_2 photodissociation / photoionization yields product channels with generally high kinetic energy, for example the most intense ring visible in each image in Figure S9 corresponds to an O^+ speed of 1600 ms^{-1}. It is therefore not appropriate for calibration over the much lower range of speeds typically relevant in rotationally inelastic scattering.

We therefore also calibrated the ion optics at a total potential of +600 V, as used in the experiments reported in this paper, using the 325 nm photodissociation of NO_2 to produce NO($X^2\Pi$, v = 1) in a range of rotational states with well-defined speeds similar to those resulting from rotationally inelastic scattering. By overlapping the low intensity probe laser pulse at ≈ 236 nm and the much more intense 325 nm ionization pulse we were able to record these images via 1 + 1′ REMPI after dissociation with the same 325 nm pulse. The dissociation has a single product channel, and the photodissociation threshold and NO energetics are very well known,[5] and hence only the distribution of spin-orbit states of the coincident O(^{3}P) provides any ambiguity in the product NO speed. These calibration experiments were performed with the NO_2 (10%) seeded in Ar and using a molecular beam propagating in the plane of the image. The resulting spread of speeds in the NO_2 before photodissociation obscured any fine structure arising from the co-incident O-atom spin-orbit state distribution.

$$NO_2 + h\nu \rightarrow NO(X, v = 1, j = 0.5\ F_1e) + O(^3P_2) \quad \text{TKER} = 3765\ \text{cm}^{-1}$$

We recorded crush images for 8 rotational levels in the F_1 spin-orbit manifold, specifically j = 15.5*f*, 20.5*f*, 25.5*f*, 30.5*f*, 35.5*f*, 38.5*e*, 44.5*f* and 45.5*f*. Example images for final states j = 15.5*f* and 30.5*f* are presented in Figure S11.

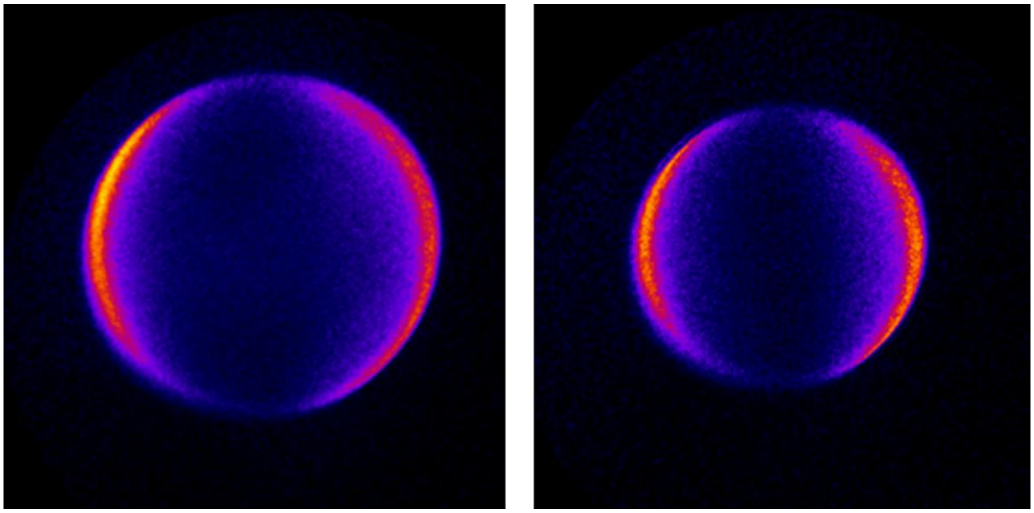

Figure S11. Example images of NO(X, v = 1, j) from the 325 nm photodissociation of NO_2. Left panel j = 15.5 F_1f, right panel j = 30.5 F_1f. The electric vector of the photolysis laser was along the x-axis of the image, and the molecular beam propagated diagonally from top right to bottom left.

Each image was Abel inverted and the diameter of the resulting NO ring is plotted against calculated NO speed, assuming co-incident $O(^3P_2)$ production, in Figure S12. The excellent linear fit results in a speed-to-pixel ratio of 2.89 ± 0.02 ms^{-1} $pixel^{-1}$ for NO at +600 V overall potential.

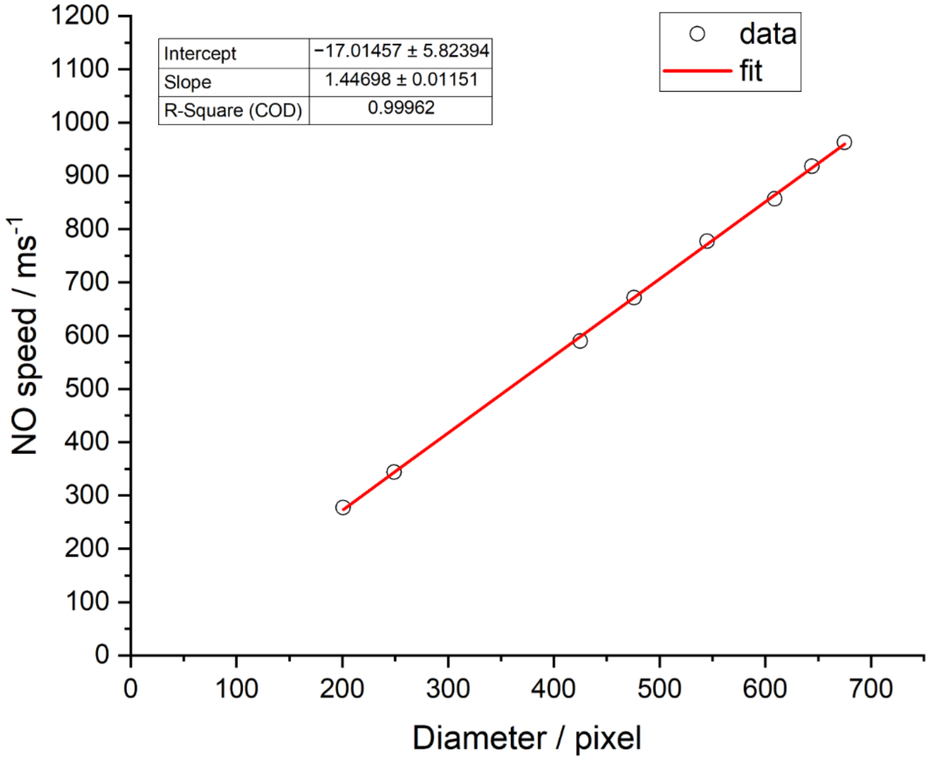

Figure S12. Speed-to-pixel calibration for *m*/*z* = 30 at a repeller potential of +600 V. Data points (open circles) from analysis of Abel-inverted images of NO (v = 1, j) from the 325 nm photodissociation of NO_2. Linear fit (red line) to determine the image calibration factor, 2.89 ± 0.02 ms^{-1} $pixel^{-1}$.

3. NO – CH_4 image fitting

We performed image fitting on the NO(X) + CH_4 results for both final state j' = 4.5 F_1f and j' = 10.5 F_1f using the software applied to the NO(X) + Ar results, to further demonstrate the degree of energy transfer to CH_4 rotation that is present in the data. In this analysis, the complete areas of both the H and V geometry images were fitted simultaneously to a linear combination of basis images to determine the DCS, assuming that no energy could be transferred to CH_4, i.e. ΔE_{CH4} = 0 cm^{-1}. The NO and CH_4 molecular beam parameters and other experimental details were unchanged from those used in the main text peeling analysis, with the polarization parameters determined by the kinematic apse model assuming ΔE_{CH4} = 0 cm^{-1}. Figure S13 shows the data and fits for both polarizations for both final states, as well as subtractions of the fits from the data. It is immediately apparent that the fit images have a much sharper outer edge for both j' = 4.5 and j' = 10.5, a consequence of the constraint applied in fixing ΔE_{CH4} = 0 cm^{-1}. Although the general form of the angular scattering is recovered, the fits in all cases do not provide an accurate representation of the radial intensity dependence. The in-filling of intensity in the data images arising from energy transfer to CH_4 rotation results in the fit over-determining the outer edge intensity and under-determining the inner image intensity in all cases. This can only arise if a significant fraction of the scattered NO is formed in coincidence with energy transfer to CH_4 rotation, as determined by the peeling fit procedure described in the main text.

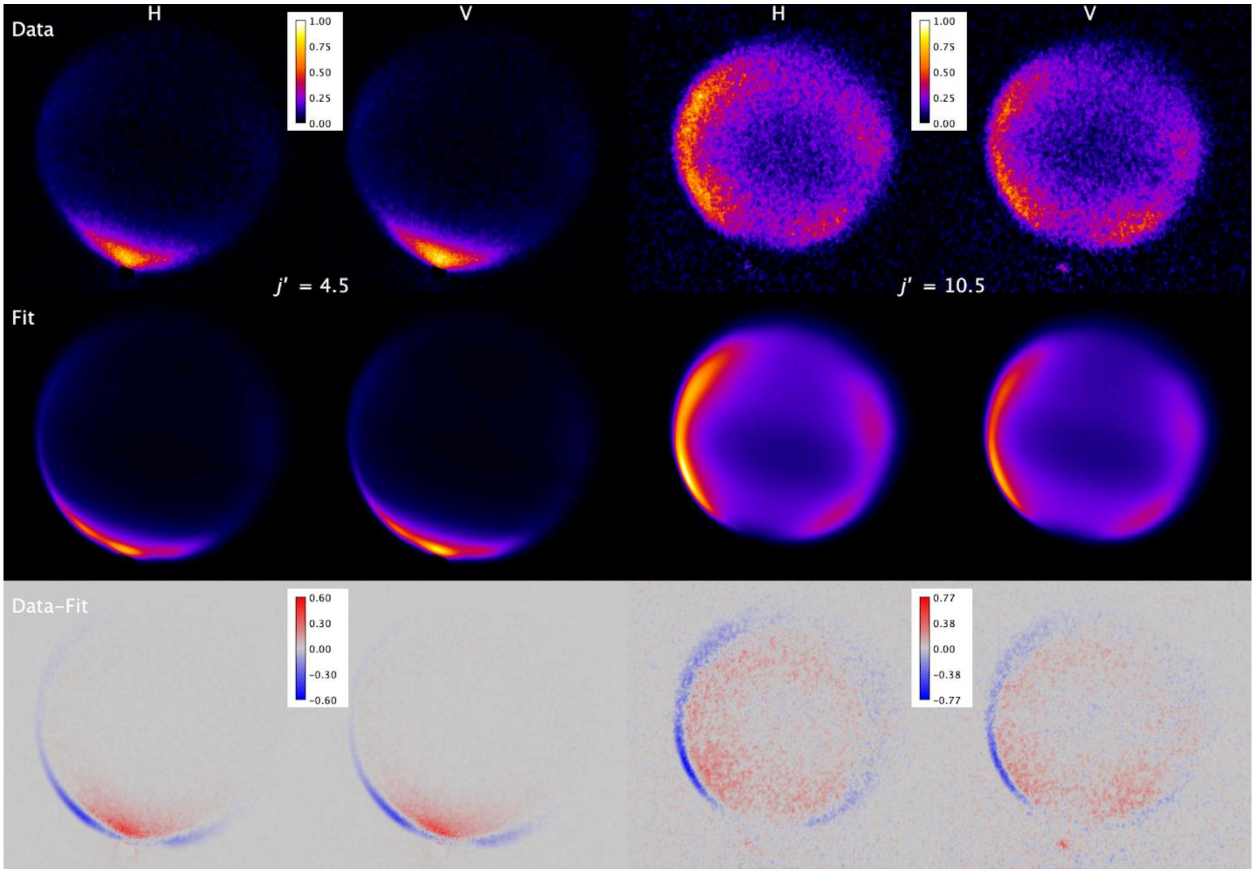

Figure S12. Data, fit and data-fit subtraction images for NO(X, v = 1, j = 1.5 F_1e) + CH_4 scattering to final states j' = 4.5 F_1f and j' = 10.5 F_1f, for both horizontal (H) and vertical (V) probe polarizations. The data is the sum of all individually acquired images, as presented in Figure 5 in the main text. The fits are the result of the NO(X) + rare gas fitting procedure, used to fit NO(X) + Ar scattering in the main text, with appropriate parameters to describe the CH_4 molecular beam and the assumption of angular momentum polarization moments consistent with kinematic apse conservation.

REFERENCES

1. Tkac, O.; Sage, A. G.; Greaves, S. J.; Orr-Ewing, A. J.; Dagdigian, P. J.; Ma, Q. L.; Alexander, M. H., Rotationally inelastic scattering of CD_3 and CH_3 with He: comparison of velocity map-imaging data with quantum scattering calculations. *Chem Sci* **2013,** *4*, 4199–4211.
2. Lin, J. J.; Zhou, J. G.; Shiu, W. C.; Liu, K. P., Application of time-sliced ion velocity imaging to crossed molecular beam experiments. *Rev Sci Instrum* **2003,** *74*, 2495–2500.

3. Eppink, A. J. B.; Parker, D. H., Velocity map imaging of ions and electrons using electrostatic lenses: Application in photoelectron and photofragment ion imaging of molecular oxygen. *Rev Sci Instrum* **1997,** *68*, 3477–3484.
4. Sun, Z. F.; Scheidsbach, R. J. A.; Banerjee, A.; Eppink, A. T. J. B.; Wei, Y. X.; Hua, Z. F.; Yang, X. Y.; Qin, Z. B.; Xu, X. S.; Zheng, X. F.; Farooq, Z.; Chen, Z. C.; Parker, D. H., Multiphoton dissociation dynamics of molecular oxygen O_2 via two-photon resonant Rydberg states in the UV region. *J Chem Phys* **2025,** *162*, 054306.
5. Jost, R.; Nygård, J.; Pasinski, A.; Delon, A., The photodissociation threshold of NO2: Precise determination of its energy and density of states. *J Chem Phys* **1996,** *105*, 1287–1290.
